# Lipid droplets and peroxisomes are co-regulated to drive lifespan extension in response to mono-unsaturated fatty acids

**DOI:** 10.1038/s41556-023-01136-6

**Published:** 2023-05-01

**Authors:** Katharina Papsdorf, Jason W. Miklas, Amir Hosseini, Matias Cabruja, Christopher S. Morrow, Marzia Savini, Yong Yu, Carlos G. Silva-García, Nicole R. Haseley, Luke Meraz Murphy, Pallas Yao, Elisa de Launoit, Scott J. Dixon, Michael P. Snyder, Meng C. Wang, William B. Mair, Anne Brunet

**Affiliations:** 1 Department of Genetics, Stanford University, Stanford, CA USA; 2 Department of Molecular Metabolism, Harvard T. H. Chan School of Public Health, Boston, MA USA; 3 Department of Molecular and Human Genetics, Huffington Center on Aging, Baylor College of Medicine, Houston, TX USA; 4 Department of Biology, Stanford University, Stanford, CA USA; 5 Glenn Laboratories for the Biology of Aging, Stanford University, Stanford, CA USA; 6 Wu Tsai Institute of Neurosciences, Stanford University, Stanford, CA USA; 7 Present Address: State Key Laboratory of Cellular Stress Biology, School of Life Sciences, Faculty of Medicine and Life Sciences, Xiamen University, Xiamen, China; 8 Present Address: Janelia Research Campus, Howard Hughes Medical Institute, Ashburn, VA USA

**Keywords:** Cell biology, Genetics

## Abstract

Dietary mono-unsaturated fatty acids (MUFAs) are linked to longevity in several species. But the mechanisms by which MUFAs extend lifespan remain unclear. Here we show that an organelle network involving lipid droplets and peroxisomes is critical for MUFA-induced longevity in *Caenorhabditis elegans*. MUFAs upregulate the number of lipid droplets in fat storage tissues. Increased lipid droplet number is necessary for MUFA-induced longevity and predicts remaining lifespan. Lipidomics datasets reveal that MUFAs also modify the ratio of membrane lipids and ether lipids—a signature associated with decreased lipid oxidation. In agreement with this, MUFAs decrease lipid oxidation in middle-aged individuals. Intriguingly, MUFAs upregulate not only lipid droplet number but also peroxisome number. A targeted screen identifies genes involved in the co-regulation of lipid droplets and peroxisomes, and reveals that induction of both organelles is optimal for longevity. Our study uncovers an organelle network involved in lipid homeostasis and lifespan regulation, opening new avenues for interventions to delay aging.

## Abstract (web summary)

Papsdorf et al. show that mono-unsaturated fatty acids extend lifespan of *C. elegans* through induction of peroxisomes and lipid droplets in fat tissues and of a lipid signature predictive of decreased lipid oxidation.

## Main

Lifespan is strongly influenced by diet. Although high-fat diets are mostly detrimental to lifespan, specific lipids can be beneficial for health and longevity^1–9^. Diets rich in mono-unsaturated fatty acids (MUFAs), such as olive oil in the Mediterranean diet, are correlated with longevity in humans^8,10^ and promote longevity in rodents^11^. Specific MUFAs (for example, oleic acid and palmitoleic acid) also causally extend the lifespan of invertebrate species such as *Caenorhabditis elegans*^7,12–16^. Yet the mechanism by which some lipids promote longevity, while others are detrimental for health, remain unclear.

Attractive candidates for the mechanism of MUFA action are conserved organelles involved in fat storage and metabolism, such as lipid droplets^17–19^. Although the role of lipid droplets has started to be evaluated during aging, age-related diseases and the response to stressors in different species^20–30^, it is still unclear whether these organelles are positive or negative regulators of health. Furthermore, the importance and mode of action of lipid droplets in MUFA-mediated longevity remain largely unknown.

## Results

### MUFA accumulation upregulates the number of intestinal lipid droplets

**Fig. 1:**
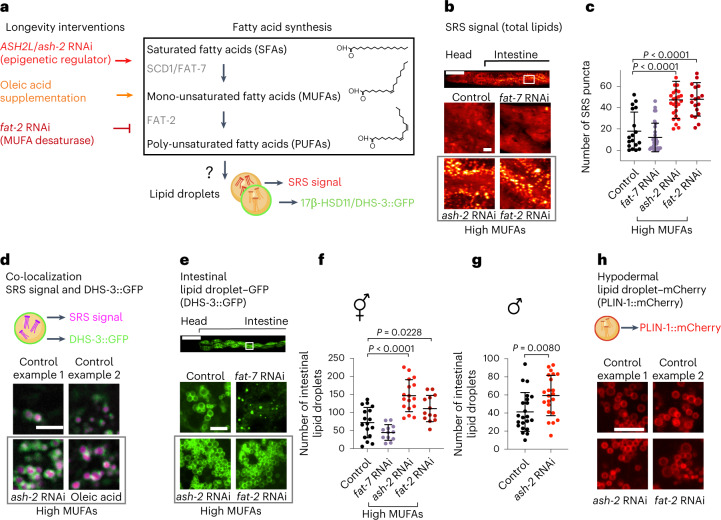
MUFAs upregulate the number of lipid droplets in the intestine. **a**, Schematic of genetic and dietary interventions that lead to MUFA accumulation in *C. elegans*. Mammalian gene names are indicated first. In all figures and panels, worms are hermaphrodites (female-like), except for Fig. 1g and Extended Data Fig. 1g, where males are used. **b**,**c**, Intestinal puncta, assessed by SRS microscopy, following MUFA accumulation. **b**, SRS image of total lipids in the anterior part of one worm (head and intestine; top). Zoomed-in images of the intestine (bottom). Scale bars, 100 µm (top) and 5 µm (bottom). **c**, Number of intestinal puncta in *n* = 18, 30, 27 and 19 worms treated with control, *fat-7*, *ash-2* and *fat-2* RNAi, respectively. Puncta intensities are provided in Extended Data Fig. 1e. **d**, Intestinal lipid droplets, assessed by SRS microscopy, in worms expressing the lipid droplet protein DHS-3 fused to GFP driven by the endogenous *dhs-3* promoter (intestinal expression; *dhs-3p::dhs-3::GFP*) following MUFA accumulation. Zoomed-in images of the intestine. Scale bar, 5 µm. Lipid droplet numbers, as assessed by double-positive puncta, are provided in Extended Data Fig. 1f. **e**,**f**, Number of intestinal lipid droplets, assessed by fluorescence, in *dhs-3p::dhs-3::GFP* worms following MUFA accumulation. **e**, Fluorescence image of the anterior part of one worm (head and intestine; top). Zoomed-in fluorescence images of the intestine (bottom). Scale bars, 100 µm (top) and 5 µm (bottom). **f**, Number of lipid droplets in *n* = 17, 12, 17 and 13 worms treated with control, *fat-7*, *ash-2* and *fat-2* RNAi, respectively. **g**, Number of intestinal lipid droplets, assessed by fluorescence, in *dhs-3p::dhs-3::GFP* worms (*n* = 22 male worms for each condition) following MUFA accumulation. **h**, Hypodermal lipid droplet number—assessed by fluorescence in worms expressing the lipid droplet protein PLIN-1 fused to mCherry driven by the endogenous *plin-1* promoter (ubiquitous expression; *plin-1p::plin-1::mCherry*)—following MUFA accumulation. Zoomed-in images of the hypodermis. Scale bar, 5 µm. Lipid droplet numbers are provided in Extended Data Fig. 1h. Elements of **a**, **d** and **h** are created with BioRender.com. **c**,**f**,**g**, Data are representative of three (**c**,**f**) or two (**g**) independent experiments. Each dot represents the number of puncta in a 26 × 26 µm^2^ area in the intestine of an individual worm. Data are the mean ± s.d. *P* values were determined using a two-tailed Mann–Whitney test. Source data are provided. Source data

**Extended Data Fig. 1:**
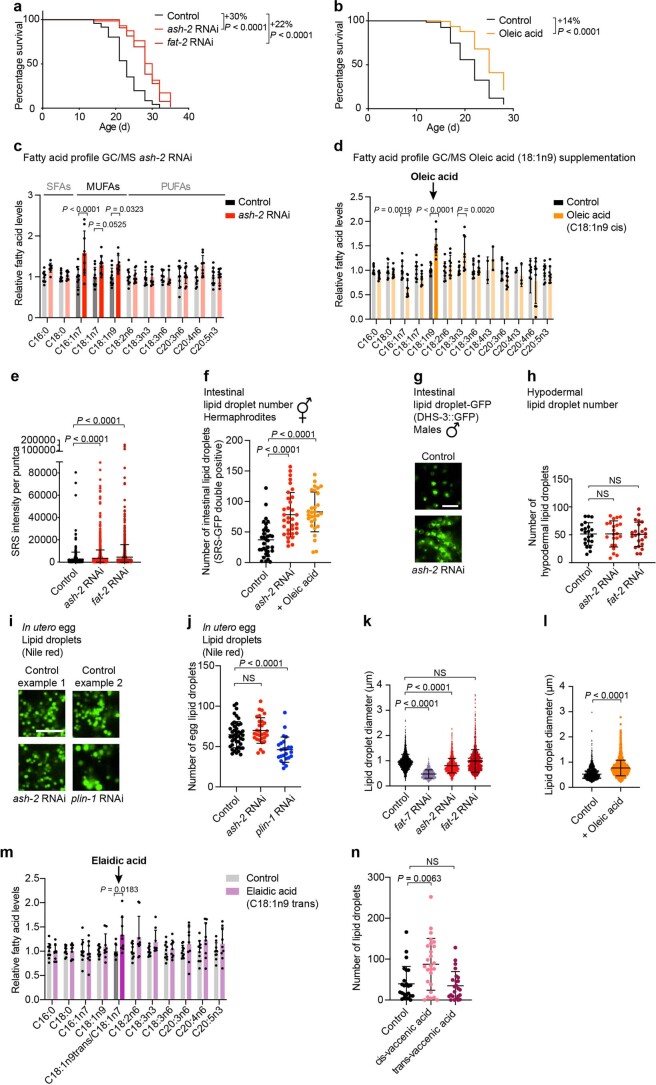
Perturbations that increase MUFAs and their effect on lipid droplets. **a**, *ash-2* or *fat-2* depletion extends lifespan; *n* ≥ 92 worms for each condition. Percentage of median lifespan extension and *P* values are indicated. *P* values: log-rank Mantel–Cox test. **b**, Dietary oleic acid supplementation extends lifespan; *n* ≥ 135 worms for each condition. Analysis as in **a**. **c**, Fatty acid profile by GC–MS following *ash-2* depletion. Fatty acid levels were normalized to the control condition. Data are the mean ± s.d. of three independent experiments, each with three biological replicates. Significant *P* values are shown. *P* values: two-way ANOVA with Bonferroni’s multiple comparison test. **d**, Fatty acid profile by GC–MS following oleic acid supplementation. Analysis as in **c**. **e**, Puncta intensity, quantified by SRS microscopy; *n* = 420, 1,603 and 927 puncta in ≥18 worms treated with control, *ash-2* RNAi and *fat-2* RNAi, respectively. Data are the mean ± s.d. Each dot represents the SRS signal intensity of one puncta. Lower segment of the *y* axis displays values of 0–90,000; upper segment of the *y* axis of 90,000–200,000. *P* values: two-tailed Mann–Whitney test. **f**, Quantification of intestinal lipid droplet number (double-positive puncta), measured by SRS microscopy, in *dhs-3p::dhs-3::GFP* worms following MUFA accumulation; *n* = 33, 34 and 29 worms treated with control and *ash-2* RNAi, and following oleic acid supplementation, respectively. Data are the mean ± s.d. Each dot represents the lipid droplet number in a 26 × 26 µm^2^ area in the intestine of an individual worm. *P* values: two-tailed Mann–Whitney test. Lipid droplets shown in Fig. 1d. **g**, Intestinal lipid droplets, assessed by fluorescence, in *dhs-3p::dhs-3::GFP* worms (males) following *ash-2* depletion. Zoomed-in images of the intestine. Scale bar, 5 µm. Lipid droplet number quantified in Fig. 1g. **h**, Quantification of hypodermal lipid droplet number, measured by fluorescence, in *plin-1p::plin-1::mCherry* worms following MUFA accumulation; *n* = 21, 22 and 23 worms treated with control, *ash-2* and *fat-2* RNAi, respectively. Data are the mean ± s.d. Each dot represents the lipid droplet number in a 15 × 15 µm^2^ area of an individual worm. Lipid droplets shown in Fig. 1h. *P* values: two-tailed Mann–Whitney test. **i**,**j**, Lipid droplets in eggs (in utero), assessed by Nile red fluorescence, following *ash-2* depletion. **i**, Zoomed-in images of one egg per condition. Scale bar, 5 µm. **j**, Quantification of lipid droplet number; *n* = 45, 30 and 24 eggs in ≥15 worms treated with control, *ash-2* and *plin-1* RNAi, respectively. Data are the mean ± s.d. Each dot represents the lipid droplet number in a 16 × 16 µm^2^ area of an individual egg*. P* values: two-tailed Mann–Whitney test. **k**,**l**, Lipid droplet size, measured by fluorescence, in *dhs-3p::dhs-3::GFP* worms following MUFA accumulation. **k**, Quantification of lipid droplet diameter; *n* = 1,720, 875, 1,803 and 1,550 lipid droplets in ≥14 worms treated with control, *fat-7*, *ash-2* and *fat-2* RNAi, respectively. Data are the mean ± s.d. Each dot represents the diameter of one lipid droplet. *P* values: two-tailed Mann–Whitney test. **l**, Quantification of lipid droplet diameter; *n* = 1,299 and 2,168 lipid droplets in ≥20 worms following control and oleic acid supplementation, respectively. Analysis as in **k**. **m**, Fatty acid profile by GC–MS following elaidic acid supplementation. Data are the mean ± s.d of two independent experiments, each with three biological replicates. Analysis as in **c**. **n**, Quantification of intestinal lipid droplet number, measured by fluorescence, in *dhs-3p::dhs-3::GFP* worms following dietary *cis*- or *trans*-vaccenic acid supplementation; *n* = 24, 26 and 24 worms following control, and dietary *cis*- and *trans*-vaccenic acid supplementation, respectively. Analysis as in **f**. **a**, Representative of three independent experiments. **b**, Representative of four independent experiments. **e**,**f**,**h**,**j**,**k**,**l**,**n**, Representative of two independent experiments. Source numerical data of all experiments, replicates and statistics are provided. Source data

We investigated whether MUFAs influence lipid droplets in *C. elegans*. To assess the number of lipid droplets, we first used stimulated Raman scattering (SRS)—a spectroscopy method that enables label-free imaging of lipids by visualizing carbon-hydrogen bonds^31–35^. MUFA accumulation was induced by upregulating SCD1 (FAT-7 in *C. elegans*), the enzyme that produces MUFAs^36,37^ (via knockdown of the chromatin regulator *ASH2L* (*ash-2* in *C. elegans*)^13^), or downregulating FAT-2, the enzyme that catabolizes MUFAs^13,14,37,38^ (Fig. 1a). We verified that these manipulations led to lifespan extension and MUFA accumulation (Extended Data Fig. 1a–d)^13,14,38,39^. Interestingly, MUFA accumulation by *ash-2* or *fat-2* RNA interference (RNAi) resulted in an increased number of puncta with high SRS intensity in intestinal cells, the main fat storage cells in *C. elegans* (Fig. 1b,c). These puncta were not only more numerous but also had higher intensity following MUFA accumulation (Extended Data Fig. 1e).

We next used confocal microscopy with a transgenic strain that expresses the lipid droplet membrane protein dehydrogenase-3 (DHS-3) fused to green fluorescent protein (GFP), which has been used to assess lipid droplets in *C. elegans*^40–42^. The DHS-3 protein is orthologous to 17β-HSD11 in mammals^43^. We verified that the puncta identified by SRS signal colocalized with GFP fluorescence^40^ (Fig. 1d), indicating that they are lipid droplets. Quantification of fluorescence in the DHS-3::GFP strain confirmed that MUFA enrichment increased the number of lipid droplets in the intestine, the main fat storage tissue in *C. elegans* (Fig. 1d–f and Extended Data Fig. 1f).

Both hermaphrodites (female-like) and male *C. elegans* had increased numbers of lipid droplets in the intestine following MUFA accumulation (Fig. 1g and Extended Data Fig. 1g), showing that the effect of MUFAs on the lipid droplet number in the intestine generalizes across sexes. Although MUFA accumulation increased the number of lipid droplets in the intestine, it did not affect the number of lipid droplets in the hypodermis (skin) or eggs (progeny; Fig. 1h and Extended Data Fig. 1h–j), which indicates tissue specificity for lipid droplet increase following MUFA accumulation. Finally, unlike lipid droplet number, the lipid droplet size was not affected uniformly by MUFA accumulation (Extended Data Fig. 1k,l). Thus, endogenous MUFA accumulation consistently increases the number of lipid droplets—organelles involved in lipid storage and metabolism—in the intestine of *C. elegans*.

### *Cis*-MUFA supplementation increases lipid droplet number

**Fig. 2:**
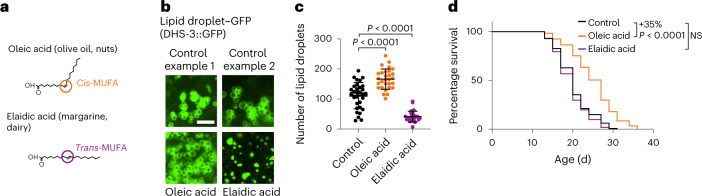
Supplementation with *cis*-MUFA (oleic acid) but not *trans*-MUFA (elaidic acid) increases lipid droplet number and extends lifespan. **a**, Chemical structure of the *cis*-MUFA oleic acid and the *trans*-MUFA elaidic acid. **b**,**c**, Number of intestinal lipid droplets, assessed by fluorescence, in *dhs-3p::dhs-3::GFP* worms following supplementation with sterically different dietary MUFAs. **b**, Zoomed-in images of the intestine. Scale bar, 5 µm. **c**, Number of lipid droplets in *n* = 31, 29 and 28 worms following control, dietary oleic acid and dietary elaidic acid supplementation, respectively. Data are the mean ± s.d. Each dot represents the number of puncta in a 26 × 26 µm^2^ area in the intestine of an individual worm. *P* values were determined using a two-tailed Mann–Whitney test. **d**, *Cis*-MUFA (oleic acid), but not *trans*-MUFA (elaidic acid), extends lifespan; *n* ≥ 128 worms for each condition. Percentages of the median lifespan extension and *P* values (log-rank Mantel–Cox test) are indicated; NS, not significant. **c**,**d**, Data are representative of three (**d**) or four (**c**) independent experiments. Source data are provided. Source data

We investigated how dietary supplementation of MUFAs impacts the number of lipid droplets. Dietary supplementation with oleic acid, a *cis*-MUFA present in olive oil and nuts, upregulated the intestinal lipid droplet number and extended lifespan (Fig. 2a–d). In contrast, dietary supplementation with elaidic acid, a *trans*-MUFA present in margarine and dairy known to have detrimental effects on human health^44^, decreased the number of lipid droplets and did not extend lifespan (Fig. 2a–d, Extended Data Fig. 1m). In agreement with this, *cis*-vaccenic acid (a *cis*-MUFA that extends lifespan^13^), but not *trans*-vaccenic acid, increased the lipid droplet number (Extended Data Fig. 1n). Hence, *cis*-MUFAs (but not *trans*-MUFAs) increase the number of lipid droplets, which correlates with lifespan extension.

### Increased lipid droplet number is critical for longevity

**Fig. 3:**
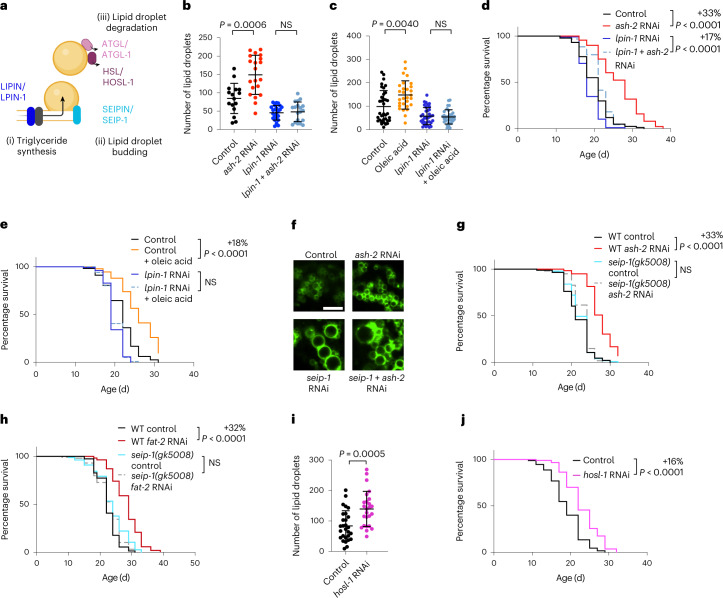
Increased lipid droplet number is necessary for MUFA-induced longevity and sufficient to extend lifespan. **a**, Schematic of conserved proteins involved in lipid droplet synthesis and degradation. Mammalian protein names are indicated first. Created with BioRender.com. **b**,**c**, Number of intestinal lipid droplets, measured by fluorescence, in *dhs-3p::dhs-3::GFP* worms following *lpin-1* depletion and MUFA accumulation. **b**, Number of lipid droplets in *n* = 16, 20, 23 and 19 worms treated with control, *ash-2*, *lpin-1*, and *ash-2* + *lpin-1* RNAi, respectively (Extended Data Fig. 2c for the efficiency of the double knockdown). **c**, Number of lipid droplets in *n* = 35, 29, 35 and 32 worms treated with control or dietary oleic acid supplementation in the absence or presence of *lpin-1* RNAi, respectively. **d**, *lpin-1* is necessary for longevity following *ash-2* depletion; *n* ≥ 96 worms for each condition. **e**, *lpin-1* is necessary for longevity following dietary supplementation with oleic acid; *n* ≥ 105 worms for each condition. **f**, Number of intestinal lipid droplets, assessed by fluorescence, in *dhs-3p::dhs-3::GFP* worms following *seip-1* and *ash-2* depletion. Zoomed-in images of the intestine. Scale bar, 5 µm. Lipid droplet numbers are provided in Extended Data Fig. 2e. **g**, *seip-1(gk5008)* is necessary for longevity following *ash-2* depletion; *n* ≥ 64 worms for each condition. **h**, *seip-1(gk5008)* is necessary for longevity following *fat-2* depletion; *n* ≥ 90 worms for each condition. **i**, Number of intestinal lipid droplets, measured by fluorescence, in *dhs-3p::dhs-3::GFP* worms following *hosl-1* depletion; *n* = 28 and 25 worms treated with control and *hosl-1* RNAi, respectively. **b**,**c**,**i**, Each dot represents the number of lipid droplets in a 26 × 26 µm^2^ area of the intestine of an individual worm. Data are the mean ± s.d. *P* values were determined using a two-tailed Mann–Whitney test. **j**, *hosl-1* depletion is sufficient to extend lifespan; *n* ≥ 94 worms for each condition. **d**,**e**,**g**,**h**,**j**, Percentages of median lifespan extension and *P* values are indicated. *P* values were determined using a log-rank Mantel–Cox test. **b**–**e**,**g**–**j**, Data are representative of three (**b**–**e**,**g**,**i**,**j**) or two (**h**) independent experiments. NS, not significant; WT, wild-type worms. Source data are provided. Source data

**Extended Data Fig. 2:**
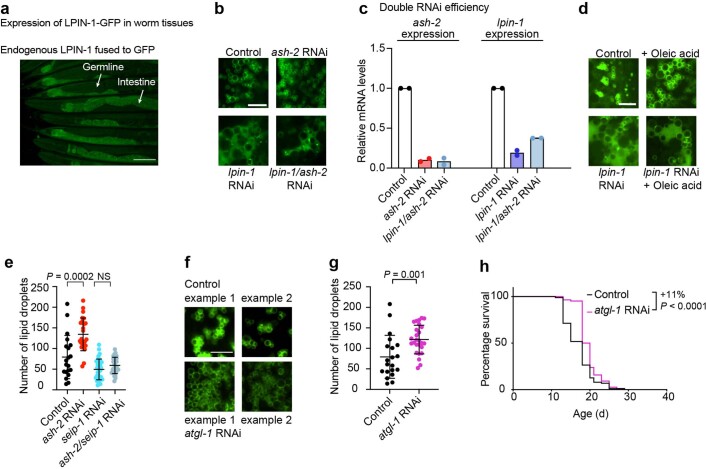
Lipid droplet perturbations that are important for longevity. **a**, Endogenous LPIN-1 expression, measured by fluorescence of GFP tagged to the C-terminus of LPIN-1 at the endogenous *lpin-1* locus. Fluorescent images of the anterior and posterior parts of young adult *lpin-1(wbm76[lpin-1::GFP])* worms. Scale bar, 100 µm. **b**, Intestinal lipid droplets, assessed by fluorescence, in *dhs-3p::dhs-3::GFP* transgenic worms following *lpin-1* and *ash-2* depletion. Zoomed-in images of the intestine. Scale bar, 5 µm. Lipid droplet number is quantified in Fig. 3b. **c**, RNAi efficiency in conditions using two RNAi constructs. RT–qPCR on RNA extracted from worms treated with control, *ash-2*, *lpin-1* and *ash-2* + *lpin-1* RNAi. The mRNA levels of target genes relative to *act-1* mRNA levels were normalized to the empty vector controls. Data are the mean of two independent experiments, with three replicates each. **d**, Intestinal lipid droplets, assessed by fluorescence, in *dhs-3p::dhs-3::GFP* transgenic worms following *lpin-1* depletion and oleic acid supplementation. Zoomed-in images of the intestine. Scale bar, 5 µm. Lipid droplet number is quantified in Fig. 3c. **e**, Quantification of intestinal lipid droplet number, measured by fluorescence, in *dhs-3p::dhs-3::GFP* worms following *seip-1* and *ash-2* depletion; *n* = 20, 25, 31 and 28 worms treated with control, *ash-2*, *seip-1*, and *ash-2* + *seip-1* RNAi, respectively. Data are the mean ± s.d. Each dot represents the lipid droplet number in a 26 × 26 µm^2^ area in the intestine of an individual worm. *P* values: two-tailed Mann–Whitney test. Lipid droplets shown in Fig. 3f. **f**,**g**, Intestinal lipid droplet number, assessed by fluorescence, in *dhs-3p::dhs-3::GFP* worms following *atgl-1* depletion. **f**, Zoomed-in images of the intestine. Scale bar, 5 µm. **g**, Quantification of lipid droplet number; *n* = 20 and 26 worms treated with control and *atgl-1* RNAi, respectively. Analysis as in **e**. **h**, *Atgl-1* knockdown extends lifespan; *n* ≥ 108 worms for each condition. The percentage of median lifespan extension and *P* values are indicated. *P* values: log-rank Mantel–Cox test. **a**,**e**,**h**, Representative of two independent experiments. **g**, Representative of three independent experiments. Source numerical data of all experiments, replicates and statistics are provided. Source data

We investigated whether the increase in the number of lipid droplets is necessary for MUFA-induced lifespan extension. Lipid droplet organelles are generated by a process involving conserved proteins (Fig. 3a). We targeted *LIPIN1* (*lpin-1* in *C. elegans*), which is important for lipid droplet synthesis^45–50^ (Fig. 3b), among other functions in lipid homeostasis^51–54^, and is expressed in the intestine (Extended Data Fig. 2a). *lpin-1* RNAi knockdown resulted in fewer lipid droplets in basal conditions and prevented the increase in lipid droplets due to MUFA accumulation (Fig. 3b,c and Extended Data Fig. 2b,d). Interestingly, *lpin-1* deficiency blunted longevity by MUFA accumulation due to *ash-2* depletion (Fig. 3d) or oleic acid supplementation (Fig. 3e).

We also targeted *SEIPIN* (*seip-1* in nematodes), which is implicated in the early steps of lipid droplet biogenesis^55–59^. *seip-1* knockdown abolished the increase in lipid droplet number in response to MUFA accumulation by *ash-2* RNAi (and led to heterogeneous lipid droplets, with a few very large droplets and some small droplets; Fig. 3f and Extended Data Fig. 2e). Consistent with these data, *seip-1* mutants no longer exhibited lifespan extension in response to MUFA accumulation by *ash-2* or *fat-2* RNAi (Fig. 3g,h). Thus, an increase in the number of lipid droplets is necessary for MUFA-induced longevity; however, other lipid droplet characteristics (for example, heterogeneity) or other aspects of lipid metabolism could also contribute.

Conversely, depletion of *HSL* (*hosl-1* in nematodes) or *ATGL* (*atgl-1* in nematodes), which are involved in lipid droplet hydrolysis (Fig. 3a)^19,60–62^, led to an increase in lipid droplets and a slight but significant lifespan extension (Fig. 3i,j and Extended Data Fig. 2f–h). Hence, an increase in the number of lipid droplets is sufficient to extend lifespan, suggesting a beneficial impact of this organelle on lifespan.

### Lipid droplet number predicts remaining lifespan

**Fig. 4:**
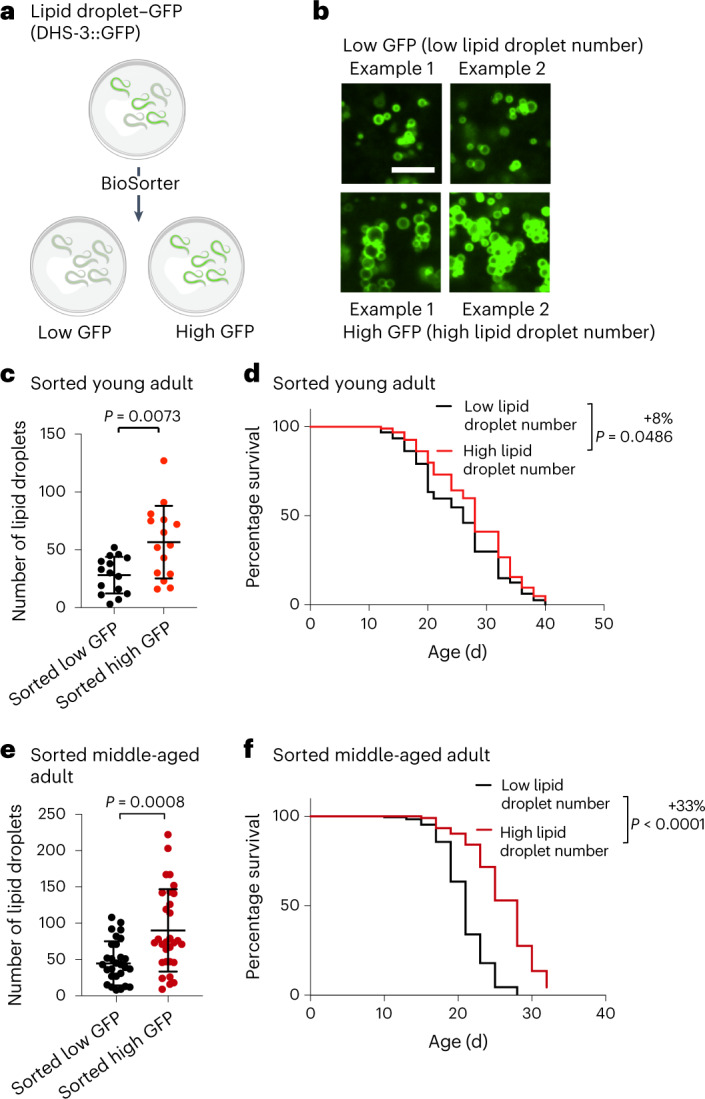
Increased numbers of lipid droplets in young or middle-aged individuals is predictive of a long life. **a**, Experimental set-up for sorting worms according to the fluorescence intensity of the DHS-3::GFP lipid droplet reporter in *dhs-3p::dhs-3::GFP* worms using a large-particle BioSorter. Created with BioRender.com. **b**,**c**, Number of intestinal lipid droplets, assessed by fluorescence, in a synchronized population of young adult (adult day 1) *dhs-3p::dhs-3::GFP* worms after sorting using a BioSorter. **b**, Zoomed-in images of the intestine. Scale bar, 5 µm. **c**, Number of lipid droplets in *n* = 15 worms for each condition. **d**, Worms sorted at young-adult age (adult day 1) with high numbers of lipid droplets live longer than worms with low numbers of lipid droplets; *n* ≥ 117 worms for each condition. **e**, Number of intestinal lipid droplets, measured by fluorescence, in a synchronized population of middle-aged (adult day 6) *dhs-3p::dhs-3::GFP* worms after manual sorting; *n* = 30 worms for each condition. Zoomed-in images are provided in Extended Data Fig. 3b. **c**,**e**, Each dot represents the number of lipid droplets in a 26 × 26 µm^2^ area of the intestine of an individual worm. Data are the mean ± s.d. *P* values were determined using a two-tailed Mann–Whitney test. **f**, Worms sorted at middle age (adult day 6) with high numbers of lipid droplets live longer than worms with low numbers of lipid droplets; *n* ≥ 195 for each condition. **d**,**f**, Percentages of the median lifespan extension and *P* values, determined using a log-rank Mantel–Cox test, are indicated. **c**–**f**, Data are representative of two (**c**,**d**) or three (**e**,**f**) independent experiments. Source data are provided. Source data

**Extended Data Fig. 3:**
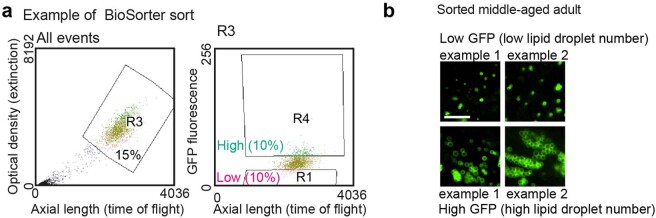
BioSorter gating strategy and sorting example. **a**, BioSorter sorting example on a synchronized population of adult *dhs-3p::dhs-3::GFP* worms according to their GFP fluorescence intensity (as a proxy for lipid droplet number). Optical density (extinction) and axial length (time of flight) of all particles measured by the sorter (left). Gate R3 was used to exclude bacterial debris and eggs and enrich for adult worms (15% of all particles). Individual worms from Gate R3 were sorted by GFP fluorescence intensity to separate the highest 10% fluorescent worms (Gate R4; right) and the lowest 10% fluorescent worms (Gate R1; right). **b**, Intestinal lipid droplet number, assessed in a synchronized population of middle-aged (adult day 6) *dhs-3p::dhs-3::GFP* worms, after manual sorting. Zoomed-in images of the intestine. Scale bar, 5 µm. Lipid droplet number is quantified in Fig. 4e.

Could the number of lipid droplets in young or middle-aged individuals predict their remaining lifespan? To address this question, we assessed the lifespan of genetically identical individuals in a population of *C. elegans* with varying amounts of lipid droplets. We used a large-particle BioSorter^63,64^ to sort two subpopulations of young adult worms expressing high or low levels of fluorescence of lipid droplet marker fused to GFP (Fig. 4a and Extended Data Fig. 3a). We verified that the worms with higher fluorescence had more lipid droplets compared with those with lower fluorescence (Fig. 4b,c). Interestingly, young individuals with more lipid droplets lived slightly but significantly longer than their counterparts with fewer lipid droplets (Fig. 4d). The predictive power of lipid droplet number for longevity was even more evident in middle-aged individuals: middle-aged individuals with more lipid droplets lived 33% longer than their counterparts with fewer lipid droplets (Fig. 4e,f and Extended Data Fig. 3b). Thus, increased numbers of lipid droplets in young or middle-aged individuals predicts their remaining lifespan, corroborating the positive role of lipid droplet number for longevity.

### Lipidomics analysis of MUFA-enriched worms

**Fig. 5:**
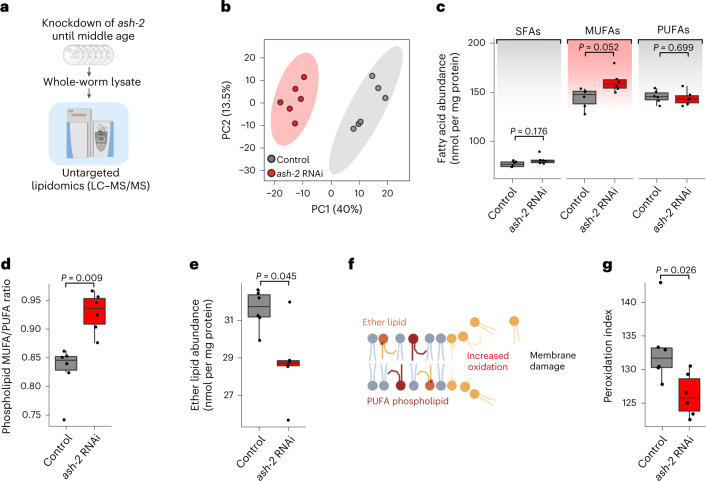
Lipidomic datasets of MUFA-enriched worms reveal changes in membrane lipids and predict decreased lipid oxidation. **a**, Untargeted lipidomic analysis on whole worms using LC–MS/MS. **b**, Principal component (PC) analysis of the lipidome separates MUFA-enriched conditions (*ash-2* RNAi) from control conditions. **c**, Fatty acyl chain abundance of saturated fatty acids (SFAs), MUFAs and PUFAs among all lipids in middle-aged worms following *ash-2* depletion. **d**, Lipidomic analysis of MUFAs and PUFAs in membrane lipids (phospholipids) following *ash-2* depletion. **e**, Lipidomics analysis of ether lipids following *ash-2* RNAi depletion. **f**, Schematic indicating that membrane lipid oxidation is generally increased in the presence of PUFA-containing phospholipids and ether lipids, particularly those present in PUFAs. Accumulation of lipid oxidation can lead to membrane damage and loss of membrane integrity. **g**, Lipidomic analysis of the peroxidation index (probability of lipid oxidation; calculation in Methods) following *ash-2* depletion. **a** and **f** are created with BioRender.com. **b**–**e**,**g**, Each dot represents a biological replicate; *n* = 6 independent biological replicates examined in one experiment. **c**–**e**,**g**, Box-and-whisker plots with the median (central line), 25th and 75th percentiles (outer lines), and minimum and maximum within 1.5× the interquartile range (whiskers) indicated. *P* values were determined using a two-tailed Wilcoxon test with Benjamini–Hochberg test for multiple hypothesis correction. Source data are provided. Source data

**Extended Data Fig. 4:**
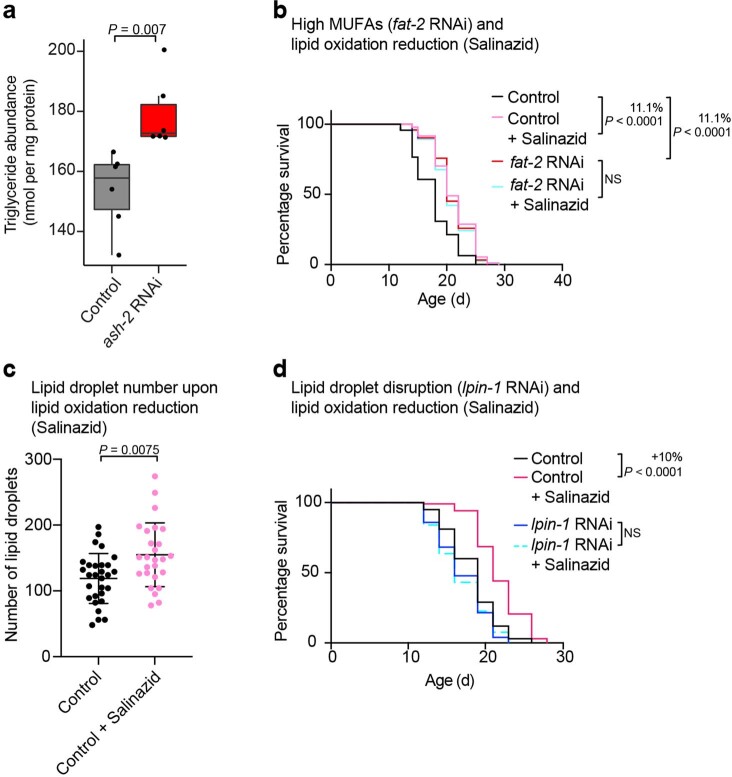
Effect of salinazid treatment on lifespan and number of lipid droplets. **a**, Triglyceride abundance among all lipids in middle-aged worms following *ash-2* depletion; *n* = 6 independent biological replicates examined in one experiment. Box-and-whisker plot, with median (central line), 25th and 75th percentile (outer lines) and minimum and maximum within 1.5× the interquartile range (whiskers). Each dot represents one biological replicate. *P* values: two-tailed Wilcoxon test with Benjamini–Hochberg test for multiple hypothesis correction. **b**, Salinazid and *fat-2* depletion act in the same pathway to extend lifespan; *n* ≥ 111 worms for each condition. Percentages of median lifespan extension and *P* values are indicated. *P* values: log-rank Mantel–Cox test. **c**, Intestinal lipid droplets, measured by fluorescence, in *dhs-3p::dhs-3::GFP* worms following Salinazid treatment; *n* = 29 and 26 worms treated with and without salinazid, respectively. Data are the mean ± s.d. Each dot represents the lipid droplet number in a 26 × 26 µm^2^ area in the intestine of an individual worm. *P* values: two-tailed Mann–Whitney test. **d**, *Lpin-1* is necessary for longevity following salinazid treatment; *n* ≥ 105 worms for each condition. Analysis as in **b**. **b**–**d**, Representative of two independent experiments. Source numerical data of all experiments, replicates and statistics as well as Cox proportional hazard interaction values are provided. Source data

To determine how MUFAs (which increase lipid droplets) affect global lipid profiles, we performed untargeted lipidomics. We isolated all lipids from middle-aged adult worms treated with control or *ash-2* RNAi (to induce MUFA accumulation and an increase in the number of lipid droplets) and performed liquid chromatography coupled to tandem mass spectrometry (LC–MS/MS; Fig. 5a). Principal component analysis on all lipid species easily separated samples with MUFA enrichment (*ash-2* RNAi) compared with controls (Fig. 5b). As expected, *ash-2* depletion led to a global increase in MUFAs and triglycerides (Fig. 5c and Extended Data Fig. 4a).

Interestingly, MUFA accumulation by *ash-2* RNAi also led to the remodeling of many membrane lipids, with an increase ratio of MUFA/poly-unsaturated fatty acid (PUFA) in membrane lipids (Fig. 5d) and a decrease in specific membrane lipids—ether lipids—which have an ether bond instead of the classical ester bond (Fig. 5e). Membrane lipids with a low MUFA/PUFA ratio and high ether lipids are associated with increased lipid oxidation (Fig. 5f)^65–68^. In agreement with this, the peroxidation index—a measure of the likelihood of lipid oxidation—was reduced in MUFA accumulation conditions (Fig. 5g). These data raise the possibility that MUFA accumulation could decrease lipid oxidation.

### MUFAs decrease lipid oxidation during aging

**Fig. 6:**
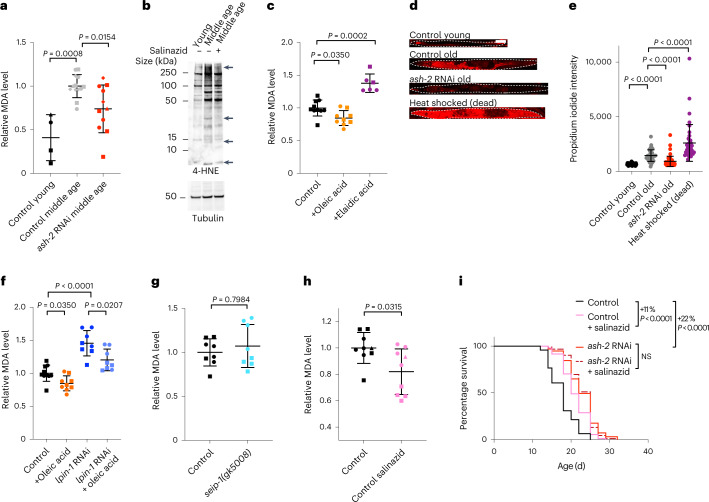
MUFAs decrease age-dependent lipid oxidation and preserve cell and membrane integrity. **a**, Relative MDA levels, used as a measure of the level of lipid oxidation, during aging and following *ash-2* depletion. Data for *n* = 4, 13 and 11 samples from control young, control middle-aged (control RNAi) and middle-aged worms treated with *ash-2* RNAi, respectively, were normalized to the middle-aged control of the corresponding experiment. **b**, Lipid oxidation assessed via western blotting for 4-HNE levels during aging and following salinazid treatment. The arrows indicate bands that change following salinazid treatment. **c**, Levels of lipid oxidation, quantified via MDA levels, following supplementation with oleic acid or elaidic acid; *n* = 10, 9 and 6 samples from middle-aged worms with control, oleic acid and elaidic acid supplementation, respectively. **d**,**e**, Cell and membrane integrity, assessed according to the intensity of propidium iodide (PI) staining, during aging and following *ash-2* depletion. **d**, Images of one worm per condition stained with PI. Dashed lines outline the worms. Scale bar, 100 µm. **e**, Intensity of PI staining for *n* = 32, 40, 40 and 55 young and old worms treated with control RNAi, old worms treated with *ash-2* RNAi and dead worms, respectively. Each dot represents the mean PI signal in one worm. **f**, Levels of lipid oxidation, quantified via MDA levels, following oleic acid supplementation and *lpin-1* depletion; *n* = 10, 9, 8 and 8 samples from middle-aged worms with control or oleic acid supplementation in the absence or presence of *lpin-1* RNAi, respectively. **g**, Levels of lipid oxidation, quantified via MDA levels, in *seip-1* mutant worms; *n* = 8 samples from middle-aged worms for each condition. **h**, Levels of lipid oxidation, quantified via MDA levels, following salinazid treatment; *n* = 9 samples from middle-aged worms for each condition. **a**,**c**,**f**–**h**, Each dot represents a biological replicate. Each shape represents an independent experiment. **a**,**c**,**f**–**h**, Data are the mean ± s.d. of four (**a**), two (**c**,**f**,**g**) or three (**h**) independent experiments. *P* values were determined using a two-tailed Mann–Whitney test. **i**, Salinazid and *ash-2* depletion act in the same pathway to extend longevity. Percentages of the median lifespan extension and *P* values, determined using a log-rank Mantel–Cox test, are indicated; *n* ≥ 110 worms for each condition. **b**,**e**,**i**, Data are representative of two (**b**) or three (**e**,**i**) independent experiments. Source data are provided. Source data

We tested whether lipid oxidation is impacted by MUFA accumulation. To quantify lipid oxidation, we measured the levels of malondialdehyde (MDA; a degradation product of oxidized lipids) and 4-hydroxynonenal (4-HNE; a degradation product of oxidized lipids that modifies proteins). With both methods we observed that middle-aged adults had higher levels of lipid oxidation compared with their younger counterparts (Fig. 6a,b), as previously reported^69^. We showed that MUFA accumulation by *ash-2* RNAi reduced lipid oxidation in middle-aged individuals (Fig. 6a). Dietary supplementation of the *cis*-MUFA oleic acid (present in olive oil), which upregulates lipid droplets and extends lifespan, also reduced lipid oxidation in middle-aged adults (Fig. 6c). In contrast, dietary supplementation of the *trans*-MUFA elaidic acid (present in margarine and dairy), which does not upregulate lipid droplets or extend lifespan, increased lipid oxidation (Fig. 6c). These data suggest that MUFAs can counter the age-dependent increase in lipid oxidation.

Lipid oxidation is associated with ferroptosis—an iron-dependent form of cell death in mammalian cells^70–76^. Although it is unclear whether bona fide ferroptosis occurs in the intestine of *C. elegans*, we observed that propidium iodide staining—which detects lack of cell and membrane integrity^77,78^—increases in older individuals (Fig. 6d,e). MUFA accumulation reduced propidium iodide staining in older individuals (Fig. 6d,e), suggesting that MUFAs preserve cell and membrane integrity during aging, perhaps by lowering lipid oxidation.

We investigated whether lipid droplet synthesis is necessary for the decrease in lipid oxidation by MUFAs. Knockdown of *lpin-1*, which decreases the number of lipid droplets, led to a strong increase in lipid oxidation in middle-aged worms in both basal conditions and oleic acid supplementation (Fig. 6f). However, deficiency in *seip-1*, which decreases the number of lipid droplets (but increases their heterogeneity, with a few very large and some very small droplets) did not affect lipid oxidation (Fig. 6g). The discrepancy between *lpin-1* and *seip-1* could be due to different roles in lipid droplet synthesis (Fig. 3a), additional function outside of lipid droplet synthesis or different effects on lipid droplet size/heterogeneity. Together, these data suggest that *lpin-1* is critical for maintaining lower levels of lipid oxidation during aging, including in response to MUFAs.

To assess the functional importance of lipid oxidation in MUFA-induced longevity, we used salinazid, a drug that chelates iron and prevents iron-induced lipid oxidation (a characteristic of ferroptosis)^69^. We verified that salinazid reduced lipid oxidation in *C. elegans* (Fig. 6b,h). Salinazid treatment extended the lifespan of *C. elegans* in control conditions (Fig. 6i), as previously shown^69^. Interestingly, salinazid did not further extend lifespan in conditions of MUFA accumulation (by *ash-2* or *fat-2* RNAi; Fig. 6i and Extended Data Fig. 4b), suggesting that MUFAs and decreased lipid oxidation are in the same pathway. Salinazid also increased the number of lipid droplets (Extended Data Fig. 4c), and this was required for salinazid to extend lifespan (Extended Data Fig. 4d). Thus, blocking iron-induced lipid oxidation is important for MUFA-induced longevity (although salinazid could also extend lifespan via other iron-dependent processes or its effect on lipid droplet number).

Hence, MUFAs impact lipid homeostasis—with a decrease in ether lipids and lipid oxidation. Maintaining low lipid oxidation could be an important component of the beneficial effects of MUFAs on longevity.

### Peroxisomes are critical for MUFA-induced longevity

**Fig. 7:**
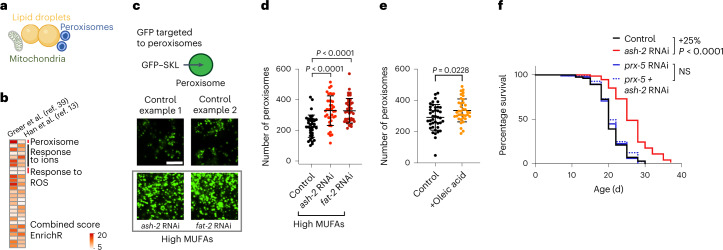
MUFAs upregulate peroxisome number, which is required for MUFA-induced longevity. **a**, Organelles such as mitochondria and peroxisomes are also involved in lipid metabolism. Created with BioRender.com. **b**, Analysis of existing transcriptomic datasets of worms following MUFA accumulation. Upregulated GO terms that are shared between worms treated with either control or *ash-2* RNAi. GO terms analyzed using WormEnrichR. GO terms upregulated in middle-aged individuals (adult day 5, whole worms; left column). GO terms upregulated in young individuals (adult day 1/intestine; right column). GO terms were considered significant if they had a combined score of >5 for the log-transformed *P* value (Fisher’s exact test) multiplied by the rank-based enrichment *z*-score. ROS, reactive oxygen species. **c**–**e**, Number of intestinal peroxisomes, assessed by fluorescence, following MUFA accumulation in worms expressing a peroxisome-localized GFP (GFP–SKL) driven by the intestinal *ges-1* promoter (*ges-1p::GFP–SKL*). **c**, Zoomed-in images of the intestine. Scale bar, 5 µm. Intensity of peroxisome-localized GFP in Extended Data Fig. 5a. **d**, Number of peroxisomes following MUFA accumulation in *n* = 39, 38 and 40 worms treated with control, *ash-2* and *fat-2* RNAi, respectively. **e**, Number of peroxisomes following dietary supplementation with oleic acid; *n* = 40 and 37 control- and oleic acid-supplemented worms, respectively. **d**,**e**, Data are the mean ± s.d. Each dot represents the number of peroxisomes in a 26 × 26 µm^2^ area in the intestine of an individual worm. *P* values were determined using a two-tailed Mann–Whitney test. **f**, *Prx-5* is necessary for longevity following *ash-2* depletion. Percentages of the median lifespan extension and *P* values are indicated; *P* values were determined using a log-rank Mantel–Cox test; NS, not significant; *n* ≥ 94 worms for each condition. **d**–**f**, Data are representative of three independent experiments. Source data are provided. Source data

**Extended Data Fig. 5:**
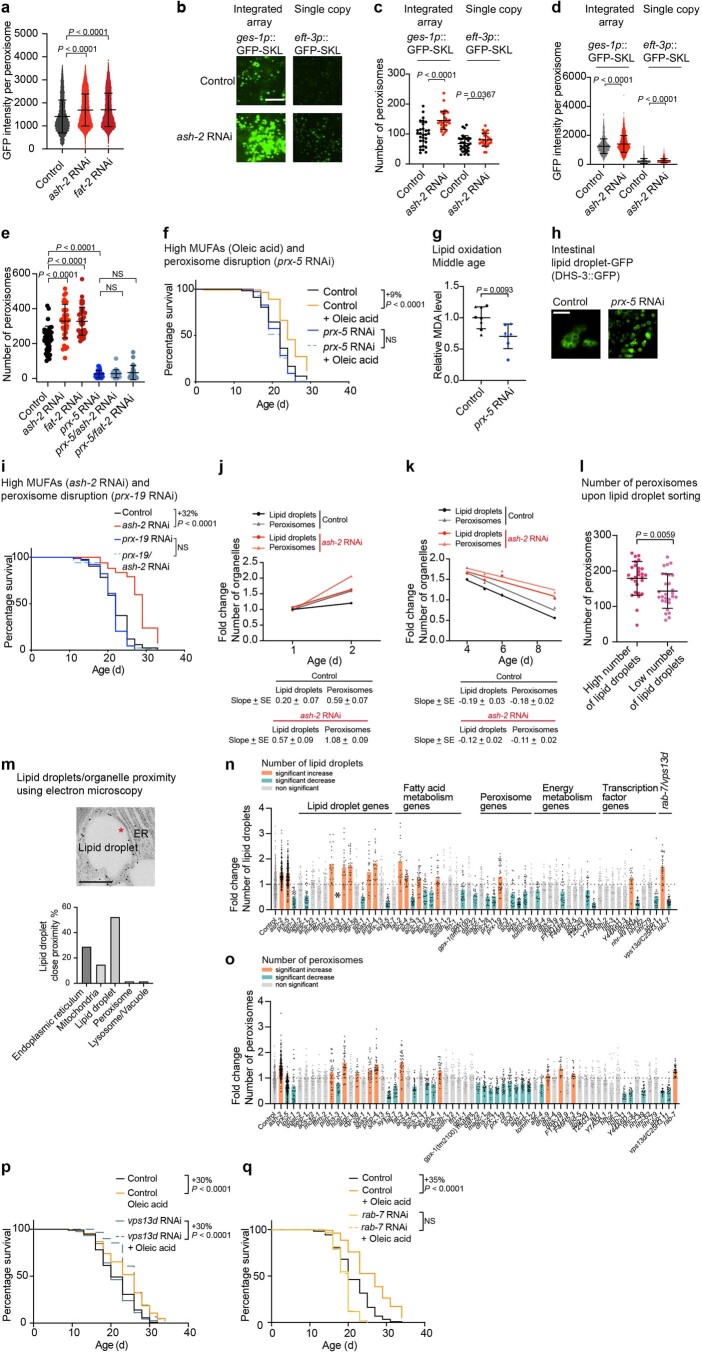
Interventions that regulate the number of peroxisomes and lipid droplets. **a**, Quantification of intestinal peroxisome-localized GFP intensity, measured by fluorescence, in *ges-1p::GFP–SKL* worms following MUFA accumulation; *n* = 6,061, 13,446 and 10,598 peroxisomes in ≥35 worms treated with control, *ash-2* and *fat-2* RNAi, respectively. Data are the mean ± s.d. Each dot represents the GFP intensity of one peroxisome. *P* values: two-tailed Mann–Whitney test. Peroxisomes shown in Fig. 7c. **b**–**d**, Intestinal peroxisomes, assessed by fluorescence, in worms expressing an integrated array of *ges-1p::GFP–SKL* or a single copy knock-in of a peroxisome-localized GFP (GFP*–*SKL) driven by the ubiquitous *eft-3* promoter (*eft-3p::GFP–SKL*) following *ash-2* depletion. **b**, Zoomed-in images of the intestine. Scale bar, 5 µm. **c**, Quantification of peroxisome number; *n* = 30, 29, 32 and 27 *ges-1p::GFP–SKL* worms treated with control and *ash-2* RNAi, and *eft-3p::GFP-SKL* worms treated with control and *ash-2* RNAi, respectively. Data are the mean ± s.d. Each dot represents the peroxisome number in a 13 × 13 µm^2^ area in the intestine of an individual worm. *P* values: two-tailed Mann–Whitney test. **d**, Quantification of peroxisome-localized GFP intensity; *n* = 1,922, 2,814, 1,537 and 1,553 peroxisomes in ≥22 *ges-1p::GFP–SKL* worms treated with control and *ash-2* RNAi, and *eft-3p::GFP–SKL* worms treated with control and *ash-2* RNAi, respectively. Analysis in **a**. **e**, Quantification of intestinal peroxisomes, measured by fluorescence, in *ges-1p::GFP–SKL* worms following *prx-5* depletion and MUFA accumulation; *n* = 39, 38, 40, 33, 26 and 22 worms treated with control, *ash-2*, *fat-2*, *prx-5*, *ash-2* + *prx-5*, and *fat-2* + *prx-5* RNAi, respectively. Data are the mean ± s.d. Each dot represents the peroxisome number in a 26 × 26 µm^2^ area in the intestine of an individual worm. *P* values: two-tailed Mann–Whitney test. **f**, *prx-5* is necessary for longevity following oleic acid supplementation; *n* ≥ 120 worms for each condition. Percentages of median lifespan extension and *P* values are indicated. *P* values: log-rank Mantel–Cox test. **g**, Lipid oxidation quantified via MDA levels following *prx-5* depletion; *n* = 8 and 7 samples from worms treated with control and *prx-5* RNAi, respectively. Normalized to the control condition. Data are the mean ± s.d. of two independent experiments. Each dot represents a biological replicate. Each shape represents an independent experiment. *P* values: two-tailed Mann–Whitney test. **h**, Intestinal lipid droplet number, assessed by fluorescence, in *dhs-3p::dhs-3::GFP* worms following *prx-5* depletion. Zoomed-in images of the intestine. Scale bar, 5 µm. Lipid droplet number quantified in n. **i**, *prx-19* is necessary for longevity following *ash-2* depletion; *n* ≥ 95 worms for each condition. Analysis as in **f**. **j**,**k**, Intestinal lipid droplet and peroxisome number, measured by fluorescence, in *dhs-3p::dhs-3::GFP*; *vha-6p::mRFP–SKL* worms during aging and following *ash-2* depletion; *n* ≥ 23 worms. Data are the mean ± s.d. Each dot represents the mean organelle number of all worms imaged for this condition normalized to young control (adult day 1) worms. Organelle number increases at younger ages (**j**) and organelle number decreases at older ages (**k**). Linear regression fit. **l**, Intestinal peroxisomes, measured by fluorescence, in *dhs-3p::dhs-3::GFP*; *vha-6p::mRFP–SKL* worms after sorting based on lipid droplet number using a BioSorter; *n* = 27 and 28 worms sorted based on high and low lipid droplet number, respectively. Analysis as in **e**. **m**, Lipid droplets assessed by immunogold labeling (against GFP) using transmission electron microscopy in *dhs-3p::dhs-3::GFP* worms. The asterisk indicates close proximity/contact between a lipid droplet and the endoplasmic reticulum (top). Scale bar, 500 nm. Quantification of lipid droplet contact/close proximity with other organelles as a percentage (bottom). **n**, Intestinal lipid droplets, measured by fluorescence, in *dhs-3p::dhs-3::GFP*; *vha-6p::mRFP–SKL* transgenic worms following treatment with 62 different RNAis; *n* = 19–423 worms. Data are the mean ± s.d. Each dot represents the organelle number in a 26 × 26 µm^2^ area in the intestine of an individual worm normalized to control worms. Orange, significant increase in organelle number. Turquoise, significant decrease in organelle number. *P* values: two-tailed Wilcoxon test with Benjamini–Hochberg test for multiple hypothesis correction. Conditions are colored if the adjusted *P* < 0.05. *dhs-3 RNAi abolishes the GFP signal of the lipid droplet DHS-3::GFP reporter. **o**, Quantification of intestinal peroxisome measured by fluorescence in *dhs-3p::dhs-3::GFP*; *vha-6p::mRFP–SKL* transgenic worms. Quantification of peroxisome number in worms treated as in **n**; *n* = 18–286 worms for each condition. Analysis as in **n**. **p**, *vps13d* depletion does not reduce longevity following oleic acid supplementation; *n* ≥ 99 for each condition. Analysis as in **f**. **q**, *rab-7* depletion reduces longevity following oleic acid supplementation; *n* ≥ 120 for each condition. Analysis as in **f**. **a**,**e**, Representative of three independent experiments. **c**,**d**,**f**,**i**–**m**,**p**,**q**, Representative of two independent experiments. Source numerical data of all experiments, replicates, exact *n* values and statistics as well as Cox proportional hazard interaction values are provided. Source data

We next investigated whether MUFAs induce other protective mechanisms that could act together with lipid droplets. Our re-analysis of transcriptomic datasets of *C. elegans* with or without MUFA accumulation^13,39^ showed peroxisome-related Gene Ontology (GO) terms in conditions of MUFA accumulation (Fig. 7a,b), raising the possibility that peroxisomes may also have a functional role in lifespan extension by MUFAs. Using a transgenic reporter strain that carries GFP fused to a peroxisome import signal^79,80^, we found that MUFA accumulation due to *ash-2* depletion, *fat-2* depletion or oleic acid supplementation led to increased intensity of the GFP signal in the intestine, indicative of increased number/function of peroxisomes in this fat storage tissue (Fig. 7c–e and Extended Data Fig. 5a–d,h). Deficiency in *PEX5* (*prx-5* in nematodes) and *PEX19* (*prx-19* in nematodes), which are critical for peroxisome function, abolished lifespan extension due to MUFA accumulation (Fig. 7f and Extended Data Fig. 5e,f,i). This effect is unlikely to be via the lowering of lipid oxidation because *prx-5* deficiency decreased lipid oxidation (Extended Data Fig. 5g). Thus, MUFAs upregulate not only lipid droplet number but also peroxisome number/function in the intestine, with peroxisome function being necessary for MUFA-induced longevity.

### Lipid droplets and peroxisomes are co-regulated

**Fig. 8:**
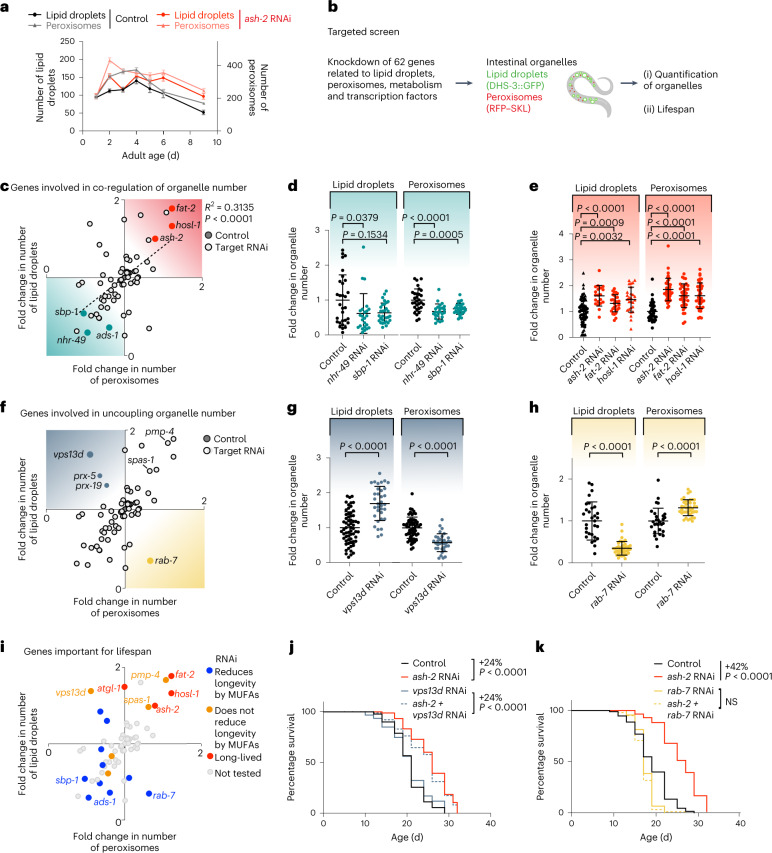
Targeted screen to identify genes involved in the co-regulation of lipid droplet and peroxisome number. **a**, Number of intestinal lipid droplets and peroxisomes, measured by fluorescence, in *dhs-3p::dhs-3::GFP*; *vha-6p::mRFP–SKL* worms during aging and following *ash-2* depletion. Data are the mean ± s.e.m. of *n* = 21–37 worms. Each dot represents the mean organelle number of all worms imaged for this condition. **b**, Targeted screen design. Created with BioRender.com **c**, Genes involved in the co-regulation of organelle number. Number of intestinal lipid droplets and peroxisomes, measured by fluorescence, in *dhs-3p::dhs-3::GFP*; *vha-6p::mRFP–SKL* worms following treatment with 62 different RNAis. RNAi of the indicated genes resulted in an increase (red) or decrease (teal) of both organelles; *n* ≥ 18 worms per condition. **d**, Number of intestinal lipid droplets and peroxisomes, quantified by fluorescence, in *dhs-3p::dhs-3::GFP*; *vha-6p::mRFP–SKL* worms following *sbp-1* and *nhr-49* transcription factor depletion; *n* = 24–30 worms. **e**, Number of intestinal lipid droplets and peroxisomes, quantified as in **d**, following *ash-2*, *fat-2* or *hosl-1* depletion; *n* = 20–42 worms. **f**, Genes involved in uncoupling lipid droplet and peroxisome number. RNAi of the indicated genes resulted in decreased numbers of lipid droplets and increased peroxisomes (yellow) or increased numbers of lipid droplets and decreased peroxisomes (blue). **g**, Number of lipid droplets and peroxisomes, measured as in **d**, following *vps13d* depletion; *n* = 33 or 34 worms. **h**, Number of lipid droplets and peroxisomes, measured as in **d**, following *rab-7* depletion; *n* = 29–41 worms. **d**,**e**,**g**,**h**, Data are the mean ± s.d. Each dot represents the organelle number of an individual worm normalized to control worms. *P* values were determined using a two-tailed Mann–Whitney test. **i**, Genes important for lifespan. Perturbations that affect the co-regulation of the number of lipid droplets and peroxisomes, color-coded according to their effect on lifespan and MUFA-induced longevity. **c**,**f**,**i**, Each dot represents the mean organelle number, normalized to control, of all worms imaged for this condition. A two-tailed Pearson’s *R*^2^ test was used to analyze correlation; dotted line, nonlinear fit. **j**, *vps13d* is not necessary for longevity following *ash-2* depletion; *n* ≥ 71 worms for each condition. **k**, *rab-7* is necessary for longevity following *ash-2* depletion; *n* ≥ 93 worms for each condition. **j**,**k**, Percentages of the median lifespan extension and *P* values, determined using a log-rank Mantel–Cox test, are indicated. **a**,**d**,**e**,**g**,**h**,**j**,**k**, Data are representative of three (**d**,**g**,**h**,**j**,**k**) or two (**a**,**e**) independent experiments. Source data are provided. Source data

We examined the relationship between lipid droplets and peroxisomes. Both organelles exhibited similar dynamics with age and in response to MUFA accumulation (Fig. 8a), with an increase in young adults, followed by a decrease at middle age (Extended Data Fig. 5j,k). Furthermore, the number of lipid droplets correlated with that of peroxisomes (Extended Data Fig. 5l). Spatially, lipid droplets and peroxisomes were only rarely in close proximity or direct contact with each other, as determined by electron microscopy analysis (Extended Data Fig. 5m). Hence, lipid droplets and peroxisomes are co-regulated in response to MUFAs in the organism and this co-regulation is unlikely to occur via direct contact.

To identify genes involved in the co-regulation between lipid droplets and peroxisomes, we performed a targeted RNAi screen for genetic perturbations that impact both organelles. We tested 62 genes involved in aspects of lipid metabolism or physically located on lipid droplets or peroxisomes^40,41,54,59,79,81–102^ (Fig. 8b and Extended Data Fig. 5n,o). As a readout for this targeted screen we used strains expressing fluorescent markers of lipid droplets and peroxisomes. Most gene perturbations impacted the number of lipid droplets and peroxisomes in the same manner (Fig. 8c–e). Perturbations that led to more lipid droplets (*ash-2*, *fat-2* or *hosl-1* knockdown) also resulted in an increase in peroxisomes (Fig. 8c,e). Interestingly, perturbations in conserved transcription factors that regulate lipid metabolism (*SREBP* (*sbp-1* in nematodes) and *PPAR* (*nhr-49* in nematodes)) or in the ether lipid synthesis enzyme *AGPS* (*ads-1* in nematodes) decreased the number of both lipid droplets and peroxisomes (Fig. 8c,d). Thus, lipid droplets and peroxisomes are co-regulated via transcription factors and lipid synthesis enzymes, although their mechanisms of action may be indirect.

The outliers of this targeted screen are interesting because they uncover genes that uncouple lipid droplet and peroxisome number and could be involved between these two organelles. Deficiency in *VPS13D* (*C25H3.11*/*vps13d* in nematodes) resulted in high numbers of lipid droplets but low numbers of peroxisomes (Fig. 8f,g). In mammalian cells VPS13D is a lipid transporter^101^ that regulates peroxisome biogenesis^103^. Conversely, deficiency in the gene *RAB7* (*rab-7* in nematodes) led to low lipid droplet and high peroxisome numbers (Fig. 8f,h). In mammalian cells RAB7 is an endosomal protein that can also localize to lipid droplets to regulate lipolysis^104^, among other processes^105^. In contrast, deficiency in genes involved in direct contact between lipid droplets and peroxisomes (*SPASTIN* (*spas-1* in nematodes) and *ABCD1* (*pmp-4* in nematodes))^84^ did not uncouple organelle number (and increased both organelles concomitantly; Fig. 5f). These results suggest that *vps13d* and *rab-7*, which regulate lipid transport and lipolysis, are involved in between lipid droplets and peroxisomes, although their mode of action may be indirect. Hence, lipid droplets and peroxisomes form an organelle network that is influenced by several types of regulators.

Finally, we investigated how regulators of the lipid droplet–peroxisome network impact lifespan. Perturbations that increased both organelles concomitantly (*ash-2*, *fat-2* and *hosl-1*) extended lifespan (Fig. 8i), whereas perturbations that decreased both organelles concomitantly (*sbp-1* and *ads-1*) blunted MUFA-induced longevity (Fig. 8i)^13^. Perturbations in genes involved in direct contact between lipid droplets and peroxisomes (*spas-1* and *pmp-4*)^84^ did not affect lifespan (Fig. 8i). Deficiency in *vps13d*, which increases the number of lipid droplets but decreases peroxisomes, did not extend lifespan or reduce MUFA-induced longevity (by *ash-2* RNAi or oleic acid supplementation; Fig. 8i,j and Extended Data Fig. 5p). In contrast, deficiency in *rab-7*, which decreases lipid droplets but increases peroxisomes, blunted MUFA-induced longevity (by *ash-2* RNAi and oleic acid supplementation; Fig. 8i,k and Extended Data Fig. 5q). Collectively, these data suggest that increased numbers of lipid droplets is more important than increased numbers of peroxisomes to promote longevity, but that a concomitant increase in both lipid droplets and peroxisomes is critical for the full beneficial impact of MUFAs on lifespan.

## Discussion

**Extended Data Fig. 6:**
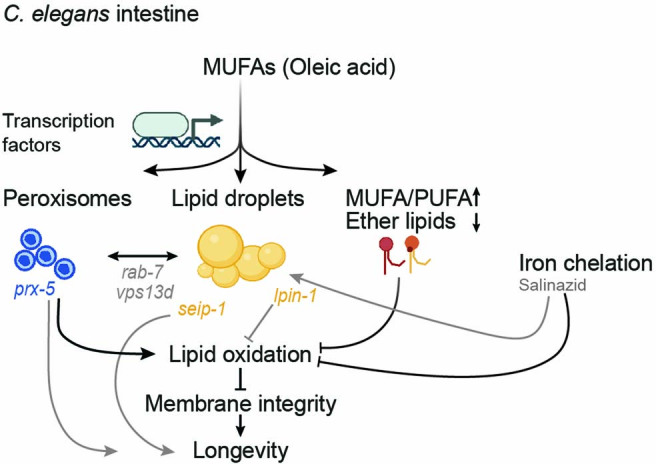
Schematic of the proposed model for how MUFAs extend lifespan. MUFA accumulation (for example, following dietary oleic acid supplementation) increases lipid droplet and peroxisome numbers, and both organelles are required for MUFA-induced lifespan extension. This concomitant increase in lipid droplets and peroxisomes requires shared upstream transcription factors (MUFAs and other lipids could also directly serve as substrates for lipid droplets synthesis). Genes encoding proteins implicated in lipid transport (*VPS13D*/*vps13d*) and organelle regulation (*RAB7*/*rab-7*) are probably involved between these two organelles (because their deficiency uncouples co-regulation of lipid droplet and peroxisome number). MUFAs increase the MUFA-to-PUFA ratio in membrane lipids and decrease ether lipids—a signature predicted to lower lipid oxidation. MUFAs reduce lipid oxidation and preserve cell and membrane integrity in the organism, and this is one way in which MUFAs extend lifespan. Genes encoding proteins that regulate lipid droplet number can also influence lipid oxidation. For example, *LIPIN1*/*lpin-1* deficiency increases lipid oxidation (but not *SEIPIN*/*seip-1*). Both *LIPIN1*/*lpin-1* and *SEIPIN*/*seip-1* deficiency reduce MUFA-induced longevity, suggesting that SEIPIN has other beneficial effects, independently of lipid oxidation, perhaps on the endoplasmic reticulum. Salinazid, an iron-chelator, prevents lipid oxidation and acts in a similar pathway as MUFAs (as these are not additive on lifespan extension). Salinazid also increases lipid droplet number, perhaps indirectly or as part of a feedforward mechanism. Peroxisome function drives lipid oxidation (probably by generating reactive oxygen species), suggesting that their beneficial effect for MUFA-induced longevity is also due to other processes (perhaps production of membrane lipids important for membrane integrity or lipids important for metabolism). Although the increase in lipid droplet number seems to be more important than that of peroxisomes, the concomitant upregulation of lipid droplet and peroxisome number in the organism is optimal for lifespan extension. Black arrows, direct effects. Gray arrows, indirect effects. Created with BioRender.com.

Our study identifies mechanisms by which dietary fatty acids such as MUFAs extend lifespan and uncovers the importance of a lipid droplet–peroxisome organelle network in longevity (Extended Data Fig. 6). We find that *cis*-MUFAs such as oleic acid (present in olive oil) increase the number of both lipid droplets and peroxisomes and modify lipid homeostasis, and that these processes are critical for MUFA-induced longevity. The magnitude of lifespan extension by MUFAs is approximately 20–30%, which is in the range of other dietary manipulations that impact the lifespan of *C. elegans* and other species^106,107^. Interestingly, the concomitant increase in the number of lipid droplets and peroxisomes is optimal for the full beneficial effects of MUFAs on lifespan.

The role of lipid droplets in health has remained unclear, with studies reporting either beneficial or detrimental effects. In *Drosophila*, overexpression of a protein that tethers lipid droplets is associated with a high median lifespan^25^ and lipid droplets can protect stem cell niches^24^. Yet lipid droplets also accumulate during old age and disease^108^, and they are detrimental in many contexts^20–22,26,28,109–111^. Our results indicate that a high number of lipid droplets in the intestine is beneficial for longevity. Other lipid droplet features such as size, heterogeneity and content may be associated with detrimental effects. Moreover, as both increased fat storage and lipolysis have been associated with longevity^6,7,16,42,112–129^, there may also be an optimal level of free MUFAs for lifespan extension. Interestingly, lipid droplet numbers in youth and middle age can predict remaining lifespan. Early life events (for example, levels of reactive oxygen species^63^) could influence MUFA levels and result in different lifespan trajectories. The predictive potential of lipid droplet number on remaining lifespan is consistent with the observation that among dietary-restricted individuals (in mice)^130^—the fattest ones live the longest.

Our lipidomics data reveal that MUFA accumulation decreases ether lipids and increases the MUFA-to-PUFA ratio in membrane lipids. It will be interesting to determine whether lipid droplets are directly involved in membrane lipid metabolism (for example, phospholipid metabolism) and, if so, what mechanisms underlie this effect. Ether lipids, which are decreased following the accumulation of MUFAs, are known to modulate lipid oxidation and ferroptosis—a conserved iron-dependent form of cell death^131–135^. Furthermore, MUFAs provide protection from ferroptosis by displacing PUFAs from membrane lipids in cancer cells^136^. Although we have not directly tested ferroptosis in *C. elegans*, we find that MUFA accumulation decreases lipid oxidation and preserves membrane and cell integrity in the intestine of older individuals. Hence, a diet high in MUFAs may change the balance of ether lipids as well as the MUFA-to-PUFA ratio in cells, thereby preventing lipid oxidation and intestinal malfunction in the organism.

Our data also reveal a previously uncharacterized connection between lipid droplets and peroxisomes in longevity. Although physical interactions between both organelles had been identified in mammalian cells^84,137^, a co-regulation at the organismal level—especially in the context of aging—was not known. Our screen identifies upstream key regulators of this lipid droplet–peroxisome network as well as genes involved between lipid droplets and peroxisomes. It will be interesting to determine whether proteins encoded by these genes act directly or indirectly to regulate the lipid droplet–peroxisome network by examining their subcellular localization during aging and their specific mechanism of action. Lipid droplets have recently been found to regulate endoplasmic reticulum responses in *C. elegans*^138^, and this could also contribute to the modulation of lipid droplets and peroxisomes during aging. The lipid droplet–peroxisome network may be critical not only for longevity but also in other biological processes or disrupted in diseases. Given the conservation of lipid metabolism and organelles in other species, our findings open new avenues—including lipid-based strategies—for promoting longevity and health.

## Methods

### *C. elegans* and bacteria strains

All *C. elegans* strains (wild type and mutants) used in this study are listed in Supplementary Table 1. Worms from mutant deletion strains were genotyped by PCR, and the PCR amplicon sizes were used to check for the presence of the deletion. *C. elegans* were cultured and maintained at 20 °C on standard Nematode Growth Media (NGM) plates seeded with a lawn of OP50-1 (gift from M. -W. Tan). For all experiments, the worms were cultured at 20 °C on RNAi plates seeded with the RNAi strains HT115 (DE3). All experiments were conducted on hermaphrodite worms, apart from the experiments in Fig. 1g and Extended Data Fig. 1g, which were conducted in male worms. Some strains were provided by the *Caenorhabditis* Genetics Center, which is funded by NIH Office of Research Infrastructure Programs (P40 OD010440) and the Mitani Laboratory at the Tokyo Women’s Medical University School of Medicine.

The WBM1177 strain (*wbmIs81[eft-3p::3XFLAG::GFP::SKL::unc-54 3*′ untranslated region (UTR), *wbmIs65]), which expresses a peroxisome localization sequence (SKL) fused to GFP driven by the *eft-3* promoter, was generated by microinjection using clustered regularly interspaced short palindromic repeats (CRISPR) into WBM1140 (*wbmIs65 [eft-3p::3XFLAG::dpy-10 crRNA::unc-54 3*′ *UTR*]) using the SKI LODGE system^139^. This system allows for knock-in of a single copy of the construct downstream of the *eft-3* promoter, which drives gene expression in all somatic cells and allows for ubiquitous expression of peroxisome-targeted GFP. The ABR339 strain *(lpin-1(wbm76[lpin-1::GFP]))*, which expresses endogenous LPIN-1 fused to GFP at the endogenous *lpin-1* locus, was generated by microinjection using CRISPR into wild-type worms. The CRISPR insertion was performed as described previously^140^. The following crRNA sequence was used for carboxy-terminal *lpin-1* editing: 5′-ATTGTTGCTGGCATCAAAAA-3′. Worms were genotyped by PCR; the PCR amplicon sizes were used to check for the presence of the insertion and the strain was crossed with wild-type worms to remove the *dpy-10* coinjection marker.

### RNAi

For knockdown by RNAi, worms were fed HT115 (DE3) transformed with vectors expressing double-stranded RNA against the gene of interest. The HT115 strains expressing RNAi targeting the gene of interest were obtained from the Ahringer library (gift from A. Fire). For all genes, the annotated WormBase name was used except for *C25H3.11*, for which we used *vps13d*. RNAi clones were confirmed by Sanger sequencing^141^. All experiments were performed on HT115.

To generate RNAi-expressing bacteria, a single bacterial colony was used to inoculate LB medium containing 100 µg ml^−1^ ampicillin (Sigma Aldrich). The bacteria were cultured overnight at 37 °C to stationary phase and the expression of the double-stranded RNAi was induced with 0.4 mM isopropylthiogalactoside (IPTG; Thermo Fisher) for 4 h at 37 °C. The cultures were centrifuged and the bacterial pellet resuspended in LB_Amp/IPTG_ (LB with 100 µg ml^−1^ ampicillin and 0.4 mM IPTG) at 1:30 of the initial volume. This bacterial resuspension was stored at 4 °C for no longer than 2 weeks. Concentrated bacteria were added to 6-cm or 10-cm NGM plates containing 100 µg ml^−1^ ampicillin and 0.4 mM IPTG (RNAi plates). For the knockdown experiments, worms were kept on 6-cm plates seeded with RNAi-expressing bacteria. Unless indicated otherwise, worms were fed the RNAi from egg lay onwards. For *lpin-1* and *fat-2* depletion, the RNAi treatment was initiated in young adults (adult day 1) to minimize the effects on development. For a negative control, worms were fed the empty vector (L4440) bacterial strain.

When two genes were knocked down simultaneously, the optical density at 600 nm (OD_600_) of the bacteria was adjusted to the same value (OD_600_ = 50, stationary phase) and the RNAi-expressing bacteria were mixed in a 1:1 ratio. To assess the gene knockdown efficiency in the context of single versus double RNAi, we performed reverse transcription, followed by real-time quantitative PCR (RT–qPCR) using primers to each gene of interest (Extended Data Fig. 2c) as described in the following section.

### RT–qPCR

To test the efficiency of gene knockdown in the context of single versus double RNAi, RT–qPCR was performed as described before^13^. Briefly, 300 wild-type worms were subjected to RNAi-expressing bacteria (or control bacteria) on 6-cm RNAi plates from egg lay to adult day 1. For experiments with *lpin-1* RNAi, knockdown for all conditions was initiated at the young adult age and worms were harvested at adult day 2. The worms were washed three times with M9 buffer (22 mM KH_2_PO_4_, 34 mM K_2_HPO_4_, 86 mM NaCl and 1 mM MgSO_4_) to remove residual bacteria in the worm pellet. To isolate total RNA, the worm pellets were resuspended in 500 µl TRIzol Reagent (Invitrogen) and subjected to six freeze–thaw cycles in a dry ice–ethanol bath. RNA was extracted according to the standard TRIzol procedure, resuspended in 30 µl of RNase- and DNase-free water, and quantified using a Nanodrop (Thermo Fisher). The RNA was treated with DNase (Thermo Fisher, 18068015), followed by reverse transcription using Oligo (dT)_20_ primers (Invitrogen, 18418020) and SuperScript IV Reverse Transcriptase (Invitrogen,18090010). iTaq Universal SYBR Green super mix (Bio-Rad,1725124) was used in a 20-µl reaction volume for the RT–qPCR reaction. Primers were designed to span exon–exon junctions and used at a final concentration of 250 nM. All primers are listed in Supplementary Table 2. RT–qPCR was performed using a C1000 thermal cycler (Bio-Rad). Melt curves were examined to ensure the specificity of the primers. Results were analyzed using the ΔΔ*C*_t_ method. For each biological replicate, the median *C*_t_ value of three technical replicates was analyzed. *act-1* served as the internal reference gene. Bar plots were generated using Prism 9. Experiments were performed twice independently, with three replicates each.

### SRS for lipid droplet quantification

To visualize all lipids in a label-free way, we used SRS microscopy^31,142^. Middle-aged (adult day 6) hermaphrodites, treated with control or RNAi-expressing HT115 bacteria, were mounted on a 2% agar pad, anesthetized with 50 mM sodium azide and covered with a glass coverslide for SRS imaging. The worms were imaged directly after mounting to avoid confounds from starvation on the microscope slide. For each experiment, approximately 18 worms were imaged per condition. The experimental set-up was built on an inverted microscope (IX81, Olympus). For SRS microscopy, spatially and temporally overlapped pulsed Pump (tunable from 720 to 990 nm, 7 ps, 80 MHz repetition rate) and Stokes (1064 nm, 5–6 ps, 80 MHz repetition rate, modulated at 8 MHz) beams provided by picoEMERALD (Applied Physics & Electronics) were coupled into an inverted laser-scanning microscope (FV1000 MPE, Olympus) optimized for near-infrared throughput. A ×20 air objective (0.75 numerical aperture (N.A.); UPlanSAPO, Olympus) and a ×60 water objective (1.2 N.A.; UPlanAPO/IR, Olympus) were used for imaging. After passing through the sample, the forward-travelling Pump and Stokes beams were collected in transmission by an air condenser (0.9 N.A.; Olympus) for the ×20 magnification and an oil condenser (1.4 N.A.; Olympus) for the ×60 magnification. A high OD bandpass filter (890/220; Chroma) was used to block the Stokes beam completely and transmit only the Pump beam onto a large area Si photodiode (FDS1010, Thorlabs) to detect the stimulated Raman loss signal. The output current from the photodiode was terminated, filtered and demodulated by a lock-in amplifier (HF2LI; Zurich Instruments) at 8 MHz to ensure shot noise-limited detection sensitivity. The laser power of the infrared laser and the optical parametric oscillator were set at 600 mW. For lipid imaging, CH_2_ signals from lipid droplets were imaged at 2,845 cm^−1^ in the SRS channel. These settings were used to visualize all lipids, as lipids are rich in CH_2_ bonds. For imaging with the SRS signal alone, two replicates were recorded at ×60 and the triplicate was recorded using a ×20 objective. Within one experiment, the same threshold was applied to all images and lipid droplets were quantified in a 26 × 26 µm^2^ area using the analyze particle function in Fiji version 2.0.0 (ref. ^143^). The experiment was carried out three times independently. For lipid droplet imaging with SRS and GFP (DHS-3::GFP, see the next section) together, two replicates were recorded at ×60 and the double-positive puncta (red SRS puncta surrounded by green GFP signal) in a 26 × 26 µm^2^ area were quantified manually. The experiment was carried out twice independently. The investigators were not blinded to allocation during experiments and outcome assessment. The lipid droplet numbers and intensities were plotted in Prism 9 and statistically significant differences between samples were assessed using the two-tailed unpaired nonparametric Mann–Whitney test.

### Confocal microscopy for lipid droplet quantification

To visualize lipid droplets by confocal microscopy, we used a reporter strain expressing the lipid droplet protein DHS-3 fused to GFP, which has been used as a marker of lipid droplets in several studies in *C. elegans*^96,144–146^. The DHS-3 protein is orthologous to 17β-HSD11 in mammals^43^. It is one of the most abundant proteins on lipid droplets found by mass spectrometry in *C. elegans*^40,41^ and its localization at the lipid droplet membrane was verified by fluorescence microscopy^40^. Transgenic *dhs-3p::dhs-3::GFP* (LIU1) hermaphrodites were imaged at middle age (adult day 6), unless noted otherwise. For each experiment, approximately 20 worms were imaged per condition. The worms were mounted on a 2% agar pad, anesthetized with 50 mM sodium azide and covered with a glass coverslide for imaging. The worms were imaged using a Nikon Eclipse Ti confocal microscope with a Zyla sCMOS camera (Andor) and the NIS-Elements software (AR 4.30.02, 64 bit) using the ×100 oil objective (Plan Apo, ×100; Nikon) and a 50-µm pinhole (0.8 µm optical section thickness) or a Zeiss confocal microscope (LSM900, Axio Observer) using the ×63 oil objective (Plan-Apochromat ×63/1.40 oil DIC M27), a 1 Airy Unit/45 µm pinhole (0.5 µm optical section thickness) and the Zen software (3.0, blue). The mid-intestinal region was imaged over 0.8 µm (five slices each, 0.2 µm interval for the Nikon confocal) and 0.92 µm (five slices each, 0.23 µm interval for the Zeiss confocal). The images on each confocal were taken using the same exposure time/laser power. Lipid droplet numbers were analyzed in Fiji version 2.0.0 (ref. ^143^) by generating *z*-stack projections of the individual slices, applying the same threshold to all images and manually counting the lipid droplets in a 26 × 26 µm^2^ area. The lipid droplet diameters were analyzed in Fiji version 2.0.0 (Ref. ^143^) by generating *z*-stack projections of five slices, applying the same threshold to all images, and manually measuring the diameter of all lipid droplets in focus.

To measure lipid droplets in males, transgenic *dhs-3p::dhs-3::GFP* (LIU1) males were maintained by crossing with hermaphrodites. For experiments, male worms were processed similarly as described earlier.

To measure hypodermal lipid droplets, transgenic *plin-1p::plin-1::mCherry* (LIU2) hermaphrodites were imaged at middle age (adult day 6). This reporter strain has been used to visualize hypodermal and intestinal lipid droplets^40^. For each experiment, approximately 20 worms were imaged per condition. The worms were processed similarly as described above with the following differences. To analyze hypodermal lipid droplets, the head hypodermis was imaged to avoid imaging intestinal lipid droplets. As the width of the worms is smaller in the head, a smaller area (15 × 15 µm^2^) was selected to count the number of lipid droplets.

To visualize lipid droplets in eggs in utero, Nile red staining was performed as described previously^41^. Briefly, young adult (adult day 1) wild-type hermaphrodites were fixed in 40% isopropanol for 3 min and stained with 8 µM Nile red (MP Biomedicals, 0215174450) for 2 h in the dark. For each experiment, approximately 20 worms were imaged per condition. The worms were mounted and imaged as described above. One or two fertilized eggs per worm were imaged in utero with the settings described above. The number of lipid droplets was counted in a 16 × 16 µm^2^ area using the Squassh plugin^147^ with the following settings: the background was subtracted with a rolling-ball radius of 10 pixels and the segmentation parameters were set at 0.05 regularization with a minimum object intensity of 0.15.

All experiments and lipid droplet analyses were performed in a blinded manner, unless noted otherwise. Key experiments were carried out three times independently but all experiments were carried out at least two times independently. The numbers and intensities of lipid droplets were plotted in Prism 9 and statistically significant differences between samples were assessed using the two-tailed unpaired nonparametric Mann–Whitney test.

### MUFA supplementation

To increase the level of specific fatty acids in *C. elegans*, we performed dietary supplementation experiments^13^. Briefly, fatty acid sodium derivatives (Nu-check prep) of oleic acid (C18:1n9 *cis*), elaidic acid (C18:1n9 *trans*), *cis*-vaccenic acid (18:1n7 *cis*) and *trans*-vaccenic acid (18:1n7 *trans*) were dissolved in water at 100 mM. To dissolve elaidic acid and *trans*-vaccenic acid, the solution was heated in a water bath at 50 °C for no more than 10 min. We verified by mass spectrometry that heated elaidic acid is still taken up (Extended Data Fig. 1m) and heated oleic acid retains its beneficial effect to increase lipid droplets and lifespan (Source Data for Fig. 2c and Extended Data Fig. 1b). The detergent Tergitol (NP-40, Sigma Aldrich) was added at a concentration of 0.001% to RNAi agar prior to autoclaving for all fatty acid (*cis* and *trans*) and control supplementation plates. After autoclaving, the agar media was cooled to approximately 60 °C and the fatty acid solutions were added to a final concentration of 0.8 mM. The agar media was stirred for 2 min after addition of the fatty acids to ensure even distribution. The plates were dried overnight in a dark ventilated space, stored at 4 °C and used within a month. Live bacteria were seeded on fatty acid-containing plates 24 h before worms were transferred to the plates. Although using fixed—metabolically inactive^148^—bacteria would be helpful for dietary MUFA supplementation, many experiments using MUFAs are also RNAi experiments, which require live bacteria.

For supplementation experiments, worms were kept on fatty acid-containing plates from egg lay onwards. If not noted otherwise, worms were treated with control (empty vector) RNAi. For experiments including *lpin-1* knockdown, RNAi treatment and fatty acid supplementation were started simultaneously at adult day 1 to minimize the negative effects of *lpin-1* knockdown on development.

### Lifespan assays

All *C. elegans* lifespan assays were performed at 20 °C on RNAi plates. Hermaphrodites were age-synchronized with a short 4-h egg lay using synchronized young adult (adult day 1) parents. The hermaphrodites were transferred to new plates and scored at least every other day to avoid the presence of confounding progeny. Each lifespan assay was performed with three plates of approximately 30 worms per 6-cm RNAi plates (approximately 90 worms in total). Worms were scored as censored if they crawled off the media or died following vulval rupture/internal hatching. Worms were scored as dead when they failed to move following gentle prodding with a platinum wire pick (90% Pt, 10% Ir). For lifespan curves, censored worms were included until the day of censorship. All lifespan experiments were performed in a blinded and randomized manner unless noted otherwise. Key experiments were carried out three times independently and all experiments were performed at least twice independently, often with independent investigators (as indicated in the source data). Kaplan–Meier survival curves were plotted in Prism 9. For pairwise comparison, the log-rank Mantel–Cox statistical test in Prism was used. To test if two interventions significantly interact with each other, the Cox proportional hazard test was applied using R (version 3.6.3). All lifespan statistics are provided in the source data.

### GC–MS analysis of fatty acid profiles

Targeted gas chromatography coupled with mass spectrometry (GC–MS) was performed as described before^13^ to ensure that fatty acid profiles change following *ash-2* RNAi, and oleic acid and elaidic acid supplementation. Briefly, for each condition, approximately 500 age-synchronized young adult (adult day 1) animals were collected in M9 buffer and washed three times to remove residual bacteria in the worm pellets. For fatty acid supplementation experiments, worms were transferred to empty (no food) RNAi plates for 20 min to clear the gut of residual bacteria^13^. Worm pellets were lysed by sonication and the protein concentration of the lysate was determined using a Pierce BCA protein assay kit (Thermo Scientific, 23227). The fatty acid C13:0 (NuChek Prep; dissolved in methanol) was added to each sample to serve as the internal reference control for variations introduced during the derivatization and extraction steps. Fatty acids were derivatized into their respective fatty acid methyl esters (FAMEs) by incubation in 2% H_2_SO_4_ (Sigma Aldrich) in methanol (Fisher) at 55 °C overnight. The reaction was stopped by the addition of 1.5 ml water (Fisher; MS grade). The FAMEs were extracted in 300 µl hexane (Sigma Aldrich) by vigorous vortexing and centrifugation at 188*g* for 1 min. The hexane layer containing the FAMEs was transferred into an amber GC vial (Agilent). FAME analysis was performed using an Agilent 7890A gas chromatograph equipped with an HP-5MS column and the MassHunter software (version 10.0.368). Each FAME peak was identified based on its retention time and unique fragmentation ions, and quantified using a serial dilution standard curve. The peaks and ion fragmentation patterns derived from elaidic acid and *cis*-vaccenic acid overlapped. These two fatty acids could not be uniquely identified with our current protocol, and we report both fatty acids together (Extended Data Fig. 1m).

The FAME abundance measured by GC–MS was normalized to the internal C13:0 reference control of each sample. For each sample, the FAME concentration (µg ml^−1^) was normalized to protein concentration (mg ml^−1^) as microgram of fatty acid detected per milligram of protein (µg mg^−1^). The fatty acid concentration of the interventions was normalized to the fatty acid concentration of the empty vector control. The final ratio is expressed as relative fatty acid levels in the graph. Each experiment was carried out at least three times independently. Relative fatty acid abundances were plotted using Prism 9 and statistically significant differences between samples were assessed using a two-way analysis of variance with Bonferroni’s multiple comparison test.

### Separating fluorescent worms using the BioSorter

To sort worms according to their lipid droplet number at young age (adult day 1), we separated hermaphrodites expressing the lipid droplet marker DHS-3 fused to GFP (*dhs-3p*::*dhs-3::GFP*, LIU1) according to their fluorescence intensity using the large particle BioSorter (Union Biometrica). To retrieve a large number of age-synchronized worms for the sorting procedure, approximately 5,000 eggs were laid by age-synchronized adult day 1 parent worms. After approximately 72 h at 20 °C, the eggs developed into adult worms that were collected in M9 buffer and sorted on a large-particle flow cytometer BioSorter (Union Biometrica) with a 6.5 psi sample cup pressure, 2.00 psi diverter pressure, 50% sheath flow rate, rotary valve, 8.0 ms drop width and 10.0 ms sort delay using the FlowPilot III software. These settings allowed for accurate sorting of single worms. The worms with the 10% highest and lowest fluorescence in the population were sorted using the 488-nm laser (Extended Data Fig. 3a). The worms were directly sorted onto 6-cm RNAi plates containing a bacterial lawn (HT115, empty vector). We confirmed that the sorting reflects the lipid droplet number by fluorescence confocal microscopy (Fig. 4b,c). For this, worms were mounted, imaged and lipid droplets quantified as described earlier. Each experiment was performed at least twice independently. BioSorter graphs were plotted using the FlowPilot III software and lipid droplet quantifications were plotted in Prism 9. Statistically significant differences between samples were assessed using the two-tailed unpaired nonparametric Mann–Whitney test.

### Separating fluorescent worms through manual sorting

To sort worms according to their lipid droplet number at middle age (adult day 6), we manually separated hermaphrodites expressing the lipid droplet marker DHS-3 fused to GFP (*dhs-3p*::*dhs-3::GFP*, LIU1) according to their fluorescence intensity on a fluorescence dissecting microscope. To retrieve a large number of age-synchronized worms for the sorting procedure, approximately 5,000 eggs were laid by age-synchronized adult day 1 parent worms. After approximately 72 h at 20 °C, the eggs developed into adult worms. The adult worms were washed each day during their reproductive period to separate adult worms from larvae/eggs. For this, worms were collected in M9 buffer in a 15-ml tube and allowed to settle to the bottom of the tube. The supernatant was removed, and adult worms were resuspended in 15 ml of fresh M9. This washing procedure was repeated five times and the adult worms were transferred to 10-cm RNAi plates containing a bacterial lawn (HT115, empty vector). Middle-aged worms (adult day 6) were sorted under a fluorescence dissecting microscope (Leica M165 FC) equipped with a Sola light engine (Leica) onto 6-cm RNAi plates containing a bacterial lawn (HT115, empty vector). We confirmed that the sorting reflects the lipid droplet number by fluorescence confocal microscopy (Fig. 4e). For this, worms were mounted, imaged and lipid droplets quantified as described earlier. The experiment was performed three times independently. Graphs were plotted in Prism 9. Statistically significant differences between samples were assessed using the two-tailed unpaired nonparametric Mann–Whitney test.

### Sample preparation for lipidomics

To analyze lipid composition using mass spectrometry, wild-type hermaphrodites were treated with control (empty vector) and *ash-2* RNAi until middle age (adult day 6). Each condition consists of six biological replicates. To retrieve a large number of age-synchronized worms, approximately 500 eggs were laid by age-synchronized adult day 1 parent worms per replicate plate. After approximately 72 h at 20 °C, the eggs developed into adult worms. The adult worms were washed each day during their reproductive period to separate adult worms from larvae/eggs. For this, worms were collected in M9 buffer in a 15-ml tube and allowed to settle to the bottom of the tube. The supernatant was removed, and adult worms were resuspended in 15 ml of fresh M9. This washing procedure was repeated six times and the adult worms were transferred to 6-cm RNAi plates containing a bacterial lawn (HT115, empty vector or *ash-2* RNAi). Middle-aged (adult day 6) worms were transferred to empty RNAi plates without any bacteria for 15 min to clear the gut of residual bacteria. The worms were collected in 200 µl M9 in Protein LoBind tubes (Eppendorf, 13-698-794). The worms were lysed using a pre-chilled stainless steel homogenizer (Wheaton, 357572) with 15 plunger strokes and the protein concentration of the lysate was determined using a Pierce BCA protein assay kit (Thermo Scientific, 23227). The lysate (from approximately 500 worms) was frozen on dry ice and stored at −80 °C.

### Lipid isolation for mass spectrometry

Lipids from whole-worm lysates were extracted using a biphasic separation with methyl tert-butyl ether, methanol and water^149^. LC–MS/MS grade reagents were used for lipidomics. Briefly, 298 μl of ice-cold methanol and 2 μl of internal standard (equiSPLASH, Avanti Polar Lipids, 330731) were added to 50 μl of worm lysate. The mixture was vortexed for 20 s and 1,000 μl of ice-cold methyl tert-butyl ether was added. The mixture was incubated under agitation for 30 min at 4 °C. After the addition of 250 μl water, the samples were vortexed for 1 min and centrifuged at 14,000*g* for 10 min at room temperature. The upper phase containing the lipids was collected and dried under nitrogen. The dry extracts were reconstituted with 300 μl of 9:1 methanol:toluene (Fisher Scientific) with 10 mM ammonium acetate (Sigma Aldrich) and centrifuged at 14,000*g* for 5 min. Water extracted using the same protocol was used as a blank control. Samples were randomized during lipid extraction.

### LC–MS/MS

To identify complex lipids, isolated lipids were analyzed with untargeted lipidomics using liquid chromatography coupled to a Q Exactive mass spectrometer (Thermo Fisher; LC–MS/MS). Lipids were separated using an Accucore C30 column 2.1 mm × 150 mm, 2.6 μm (Thermo Scientific, 27826-152130). The mobile phase solvents consisted of 1 mM ammonium formate and 0.1% formic acid in 60/40 acetonitrile/water (A), and 1 mM ammonium formate and 0.1% formic acid in 90/10 isopropanol/acetonitrile (B). The gradient profile used was 30% B for 3 min, 30–43% B over 5 min, 43–50% B over 1 min, 55–90% B over 9 min, 90–99% B over 9 min and 99% B for 5 min. Lipids were eluted from the column at 0.2 ml min^−1^, the oven temperature was set at 30 °C and the injection volume was 15 μl. The autosampler temperature was set at 15 °C to prevent lipid aggregation. The Q Exactive plus was equipped with a HESI-II probe and operated in full MS scan mode for all samples. MS/MS spectra were acquired in a data-dependent acquisition mode on pooled samples. The source conditions were as follows: spray voltage = 3.5 kV (ESI positive mode), vaporizer = 200 °C, capillary temperature = 375 °C, S-lens = 55.0%, SheathGas = 40, auxiliary gas = 8 and SweepGas = 0. The following acquisition settings were used: AGC (MS) = 3 × 10^6^, AGC (MS^2^) = 1 × 10^5^, maximum injection time (MS) = 200 ms, maximum injection time (MS^2^) = 50 ms, mass range = 260–1,900 Da, resolution MS = 70,000 (FWHM at *m*/*z* 200), resolution MS^2^ = 35,000 (FWHM at *m*/*z* 200), MS^2^ spectra were acquired in the top-10 ions in each cycle, isolation window = 1.0 *m*/*z*, dynamic exclusion = 12 s and normalized collision energy = 25–30. External calibration was performed using an infusion of Pierce LTQ Velos ESI positive ion calibration solution.

### Analysis of mass spectrometry results

Spectra were acquired in both positive and negative mode in a data-dependent manner. Lipid species were identified by matching the precursor ion mass to a database and the experimental MS/MS spectra to a spectral library containing theoretical fragmentation spectra using the LipidSearch software (version 4.1; Thermo Scientific)^150^. LipidSearch identifies phospholipids with fatty acid compositions due to the alignment of positive- and negative-mode liquid chromatography runs. Peaks corresponding to individual species were identified in both modes obtaining information of the polar head in positive mode and information of the fatty acyl chain in negative mode, similar to as described before^151^. The output of the LipidSearch software is available in GitHub (https://github.com/brunetlab). Further data processing was done using an in-house analysis pipeline written in R (version 3.6.3; available in GitHub at https://github.com/brunetlab). Briefly, processing for samples and spike-in standards were done in the same way. All ions for one lipid were aggregated and lipids with a signal <0 were discarded from further analysis. Lipid species were quantified using the corresponding internal standard (equiSPLASH, Avanti Polar Lipids, 330731) for each lipid class. Lipids with signals lower than 3× the blank signal were discarded. Lipids with more than 50% of missing values were discarded, and for the remaining missing values, imputation was performed. For this, a value was randomly assigned based on the bottom 5% for the corresponding lipid. Lipids were filtered for a coefficient of variance <0.5. Each sample was divided by its corresponding protein concentration to correct for sample-input variations. To calculate normalized abundance, each lipid within a sample was divided by the sample median, followed by multiplication with the global median. This resulted in a total of 499 filtered and normalized lipids belonging to 16 lipid classes. For a complete list of all lipidomic data, see Supplementary Table 3. Changes in the most abundant lipid classes—triacylglycerol (TG), phosphatidylethanolamine (PE), phosphatidylcholine (PC) and phosphatidylinositol (PI)—as well as differences in fatty acyl chain abundances are provided in the source data. The peroxidation index was used as a measure of the likelihood of lipid oxidation and was calculated using the following formula: 0.025 × (percentage of monoenoics) + 1 × (percentage of dienoics) + 2 × (percentage of trienoics) + 4 × (percentage of tetraenoics) + 6 × (percentage of pentaenoics) + 8 × (percentage of hexaenoics)^152^. Box plots were plotted using R (Version 3.6.3). Statistically significant differences between samples were assessed using the two-tailed Wilcoxon test with the Benjamini–Hochberg test for multiple hypothesis correction.

### MDA levels

To quantify lipid oxidation, we measured MDA, a product of the degradation of oxidized lipids. A lipid peroxidation kit (Abcam, ab118970), which uses a colorimetric reaction of MDA with thiobarbituric acid, was used to quantify the MDA levels in whole-worm lysates. Worms were treated with control (empty vector) and *ash-2* RNAi until middle age (adult day 5). Each condition consisted of several (2–4) biological replicates. Preparation of worms was carried out as described in ‘Lipid isolation for mass spectrometry’. The worm lysates were adjusted to the same protein concentration (100 µg µl^−1^) and MDA levels were measured according to the manufacturer’s instructions with the following exception: 50 µl lysate together with 150 µl TBA substrate solution were used, which allowed downscaling of sample sizes while still being in the range of the standard curve. Absorbance/fluorescence was measured using a Varioskan LUX spectrophotometer (Thermo Scientific). The investigators were not blinded to allocation during experiments and outcome assessment. Each experiment was performed at least twice independently. The MDA levels of samples were normalized to control (empty vector) RNAi conditions of middle-aged worms (adult day 5). Graphs from all independent experiments were plotted together using Prism 9. Each dot shape represents an independent experiment. Statistically significant differences between samples were assessed using the two-tailed unpaired nonparametric Mann–Whitney test.

### 4-HNE measurement by western blot

To measure lipid oxidation, we quantified the levels of 4-HNE, which is a product of the degradation of oxidized lipids and can be used as a proxy for lipid oxidation. 4-HNE reacts with cellular proteins and can be visualized using western blots of whole-worm lysates^69^. Briefly, a large number of worms was generated as described in ‘Lipid isolation for mass spectrometry’, with the following exceptions: worms were aged until adult days 1 and 8 and lysed by sonication for 30 s at 15 W on a Virsonic 600 Ultrasonic Homogenizer (Virtis Virsonic) to homogenize the tissues. The protein concentration of the lysate was determined using a Pierce BCA protein assay kit (Thermo Scientific, 23227). The protein (20–25 µg per sample) was loaded on a NuPAGE 4–12% bis-Tris PAGE gel and transferred onto a polyvinylidene fluoride membrane (0.45 µm). Ponceau staining was used to verify even transfer across the whole blot. The blots were blocked with 5% skim milk in PBS containing 0.1% Tween (PBST) and incubated using the following antibodies and dilutions: anti-4-HNE (1:2,000; AB5605, Millipore Sigma), anti-α-tubulin (1:10,000; T6074, Millipore Sigma), anti-goat-HRP (1:10,000; 401515, Calbiochem) or anti-mouse-HRP (1:10,000; 401215, Calbiochem) and visualized using enhanced chemiluminescence detection reagent (Amersham ECL, GE Healthcare). Each experiment was performed twice independently.

### Propidium iodide staining

To test for cell and membrane integrity, wild-type worms were stained with propidium iodide. Propidium iodide is a fluorescent molecule that only stains cells that have lost membrane integrity^78^. Hermaphrodites were age-synchronized with a short 4-h egg lay using synchronized young adult (adult day 1) wild-type parents. The worms were transferred to new plates every other day until adult day 11 to avoid the presence of confounding progeny. For each experiment, approximately 50 worms were imaged per condition. As a positive control for death, worms were heat shocked at 39 °C for 30 min. Propidium iodide staining was performed as described before^69^. Briefly, plates were prepared by mixing 2.5 µl of 0.5 mg ml^−1^ propidium iodide (BioLegend, 421301) with 50 µl of HT115 bacteria and adding this mix onto RNAi plates. Propidium iodide plates were prepared no more than 24 h before the staining and kept in the dark. Worms were stained with propidium iodide for 24 h. Live worms were prepared for imaging (except for the heat-shock control, in which dead worms were prepared for imaging) by mounting and anesthetizing them as described above. Images were taken using a Zeiss confocal microscope (LSM900, Axio Observer) with a ×10 air objective (×10: Pln Apo ×10/0.45 DIC II), a 23-µm pinhole (optical section thickness of 15.9 µm) and the Zen software (3.0, blue). Whole worms were imaged over 8 µm (three slices with a 4.02 µm interval). The same laser power was used within one experiment. The propidium iodide signal per worm was analyzed in Fiji version 2.0.0 (ref. ^143^) by generating *z*-stack projections and measuring the mean fluorescence intensity of each worm. The investigators were not blinded to allocation during experiments and outcome assessment. Each experiment was performed three times independently. The mean propidium iodide signal per worm was plotted in Prism 9 and statistically significant differences between samples were tested using the two-tailed nonparametric Mann–Whitney test.

### Prevention of lipid oxidation by iron chelation via salinazid

To inhibit lipid oxidation, we exposed worms to salinazid (LGC Standards, DRE-C16904350), a lipophilic compound that scavenges intracellular iron, thereby inhibiting iron-triggered lipid peroxidation that induces ferroptosis^69,153^. We used published protocols and concentrations for salinazid treatment^69^. Briefly, salinazid was dissolved in dimethylsulfoxide and added to autoclaved RNAi media before solidification to a final concentration of 250 µM. The plates were dried overnight in a dark ventilated space, stored at 4 °C and used within a month. Worms were transferred to salinazid and control (dimethylsulfoxide) plates at adult day 1. Lifespan and lipid oxidation were measured as described earlier.

### Re-analysis of gene expression datasets

We re-analyzed two independent gene expression datasets that were generated previously in the laboratory to test for shared GO term enrichment in conditions that lead to MUFA accumulation. First, we analyzed an RNA-sequencing dataset from the intestine of young adult (adult day 1) worms treated with *ash-2* RNAi^13^. We selected genes that had a log_2_-transformed fold enrichment larger than one (42 genes; adjusted *P* < 0.05) and analyzed enrichment for GO terms using WormEnrichR^154,155^. All categories of GO terms were included in the subsequent analyses. Second, we analyzed a microarray dataset from the entire body of middle-aged (adult day 5) worms treated with *ash-2* RNAi^39^. We selected genes that had a log_2_-transformed fold enrichment larger than one (304 genes; adjusted *P* < 0.05) and analyzed enrichment for GO terms using WormEnrichR. All categories of GO terms were included in the subsequent analyses. Significantly enriched GO terms (combined score > 5)^154–156^ that were shared between the two datasets were plotted using R (Version 3.6.3). The combined score was calculated by multiplying the *P* value retrieved from the Fisher’s Exact test with the *z*-score. For a detailed list of all GO terms, see the source data.

### Confocal microscopy for peroxisome quantification

Peroxisomes were visualized by confocal microscopy using worm strains that express a fluorophore fused to a peroxisome localization sequence (SKL). The fluorophore was either GFP for *ges-1p::GFP–SKL* (VS15) and *eft-3p::GFP–SKL* (WBM1177) or mRFP for *vha-6p::mRFP–SKL;* *dsh-3p::dhs-3::GFP* (ABR161). Transgenic worms were imaged at middle age (adult day 6), unless noted otherwise. For each experiment, approximately 20 hermaphrodites were imaged per condition. The last intestinal cells were imaged^80^ because they retain homogenous fluorophore expression with age. Worms were mounted as described earlier for lipid droplet quantification. The last intestinal cells (int9R, int9L or both) were imaged using the settings described for lipid droplet quantification. Images were processed in Fiji version 2.0.0 (ref. ^143^) by generating *z*-stack projections of five slices, applying the same threshold to all images and analyzing peroxisomes in a 26 × 26 µm^2^ area. For experiments with *eft-3p::GFP–SKL* (WBM1177), a smaller area of 13 × 13 µm^2^ was analyzed to exclude hypodermal peroxisomes. The Squassh plugin^147^ was used to quantify peroxisomes using the following settings: the background was subtracted with a rolling-ball radius of ten and the segmentation parameters were set at 0.075 regularization with a minimum object intensity of 0.05. Peroxisomes usually appeared as fluorescent puncta. On the rare occasion when the fluorophore was only cytosolic and failed to localize to peroxisomes, the plugin did not perform correctly, and these quantifications were excluded from further analysis. All experiments and analyses were performed in a blinded manner unless noted otherwise in the source data. Most experiments were performed three times independently but at least twice independently. Peroxisome number was plotted in Prism 9 and statistically significant differences between samples were tested using the two-tailed unpaired nonparametric Mann–Whitney test.

### Lipid droplet and peroxisome number as a function of age

To visualize lipid droplet and peroxisome dynamics as a function of age, we imaged worms during their adult life. Lipid droplets and peroxisomes were visualized at different ages and quantified as described in the previous section. For each experiment, approximately 24 hermaphrodites were imaged per condition. Peroxisomes were visualized by mRFP and lipid droplets by GFP in the *vha-6p::mRFP–SKL;* *dsh-3p::dhs-3::GFP* (ABR161) double-marker strain in the last intestinal cells as described above. Lipid droplet and peroxisome number was quantified using the same region of interest per worm. The investigators were not blinded to allocation during experiments and outcome assessment. Experiments were carried out twice independently. Mean organelle number and regression lines were plotted using Prism 9.

### Targeted lipid droplet and peroxisome screen

To identify regulators of the lipid droplet–peroxisome network, we performed a targeted RNAi screen. We selected genes to target for the screen based on proteins identified by mass spectrometry at the surface of lipid droplets^40,41^ and based on annotated protein function in lipid metabolism, lipid droplet biology, peroxisome biology, lipid transport and transcription factors involved in lipid metabolism. As a positive control, we used *ash-2* RNAi and *prx-5* RNAi. We measured the effect of RNAi knockdown of these genes on lipid droplet and peroxisome number at middle age (adult day 6) in approximately 30 hermaphrodites per condition. As the worms were imaged at day 6, it is possible that some of the effects we observed were due to compensatory mechanisms. Lipid droplets and peroxisomes were visualized and quantified as described earlier. The screen was performed in eight groups. Each group contained control (empty vector), *ash-2* and *prx-5* RNAi. Peroxisomes were visualized by mRFP (except for the first group) and lipid droplets by GFP in the *vha-6p::mRFP–SKL;* *dsh-3p::dhs-3::GFP* (ABR161) double-marker strain. For the first group, peroxisomes were measured using the single marker strain *ges-1p::GFP–SKL* (VS15) and the corresponding lipid droplet number was from an independent experiment. For all other groups, lipid droplets and peroxisomes were quantified using the same 26 × 26 µm^2^ region of interest per worm in the last intestinal cells. Organelles were quantified as described above and normalized to the control RNAi within the corresponding group. All experiments and analyses were performed in a blinded manner. To assess correlation, the mean number of organelles normalized to the control RNAi was plotted using Prism 9 and statistically significant correlation was tested using the two-tailed Pearson’s *R*^2^ test. Manhattan graphs were plotted using Prism 9 and statistical significance was tested using a two-tailed Wilcoxon test with Benjamini–Hochberg correction for multiple hypothesis correction.

### Transmission electron microscopy

We performed transmission electron microscopy to visualize cellular organelles. To ensure we identified the organelles correctly, we immunostained lipid droplets with gold antibody (see the representative image in Extended Data Fig. 5m). To this end, middle-aged (adult day 6) *vha-6p::mRFP–SKL;* *dsh-3p::dhs-3::GFP* (ABR161) hermaphrodites were fixed using a Leica ICE high pressure freezer (Leica) by placing them in a Type A, 200 µm deep, 6 mm diameter, gold-coated specimen carrier (Leica, 6770181) and covered with a Type B, 300 µm deep, 6 mm diameter aluminum specimen carrier (Leica, 16770127). The worms were picked along with enough bacteria to fill the carrier. Once frozen, carriers with worms were placed into cryo-vials with frozen fixative (1% uranyl acetate and 1% glutaraldehyde in acetone). For embedding the worms in resin, the samples were placed into a paused, pre-cooled/programmed Leica AFS unit at −50 °C, washed with acetone and then stepwise infiltrated with Lowicryl HM20 (EMSdiasum, 14345), followed by exposure to only Lowicryl HM20 (EMSdiasum, 14345). Individual specimen carriers were placed into cooled flat-bottomed TAAB plastic capsules (EMSdiasum, 70021), filled with cold Lowicryl HM20 (EMSdiasum, 14345), covered and exposed to ultraviolet light to polymerize the resin. Next, 80-nm sections were prepared using an UC7 ultramicrotome (Leica) and transferred to nickel grids. The samples were rehydrated in PBST, blocked in 0.5% ovalbumin, followed by an incubation in antibody to GFP (1:100; Abcam, ab6556) and by protein A gold (20 nm; 1:50; Sigma Aldrich, P6855). The samples were fixed in 8% glutaraldehyde, contrast stained for 40 s in 3.5% uranyl acetate in 50% acetone, followed by Sato’s lead citrate for 2 min. The sections were observed using a JEOL JEM-1400 120 kV (Jeol USA) microscope and images were taken using a Gatan OneView 4 k × 4 k digital camera (Gatan). For quantification of organelle proximity, we also used images of non-immunostained wild-type worms. To this end, middle-aged (adult day 6) wild-type hermaphrodites were fixed and sectioned^157^. The experiment was performed twice independently. Organelles in close proximity/direct organelle contacts were identified manually and plotted as bar graphs using Prism 9.

### Statistics and reproducibility

Parameters such as the minimum *n* value, mean ± s.d. and significant *P* values are reported in the figures, figure legends and source data. Significance was defined as *P* < 0.05. Pairwise comparisons were made using the Mann–Whitney test. When more than five comparisons were made, Benjamini–Hochberg correction was used for multiple hypothesis correction. When testing for correlation, Pearson’s correlation test was used, and normality distribution was confirmed using the Kolmogorov–Smirnov test. For pairwise comparison of lifespan data, the log-rank Mantel–Cox statistical test was used. To test if two lifespan interventions significantly interact with each other, the Cox proportional hazard test was applied using R (version 3.6.3). All statistics are reported in the source data. Statistical analyses were performed using Prism 9 or R (Version 3.6.3).

No statistical method was used to pre-determine sample size. No data were excluded from analysis, except for the peroxisome imaging, as described earlier. The lifespan and lipidomics assays were randomized, all other experiments were not randomized. The investigators were blinded to allocation during experiments and outcome assessment for most experiments (detailed list in the source data). Quantification and statistics were performed within a single experiment, except for GC–MS fatty acid, relative messenger RNA and MDA quantifications, which were analyzed across all replicates. Most assays were performed in three independent experiments unless specified in the corresponding method section and source data.

### Reporting summary

Further information on research design is available in the Nature Portfolio Reporting Summary linked to this article.

## Online content

Any methods, additional references, Nature Portfolio reporting summaries, source data, extended data, supplementary information, acknowledgements, peer review information; details of author contributions and competing interests; and statements of data and code availability are available at 10.1038/s41556-023-01136-6.

## Supplementary information

Reporting Summary

Supplementary Table 1 Supplementary Tables 1–3.

## Data Availability

Raw lipidomic files are available at Metabolomics Workbench (https://www.metabolomicsworkbench.org/) under the study ID ST002504. All other lipidomic files are available in the GitHub repository for this paper (https://github.com/brunetlab). For genes used in this study, the annotated WormBase name was used (www.wormbase.org). Source data are provided with this paper. All other data supporting the findings of this study are available from the corresponding author on reasonable request.

## Source data

Source Data Fig. 1 Statistical source data Fig. 1.
Source Data Fig. 2 Statistical source data Fig. 2.
Source Data Fig. 3 Statistical source data Fig. 3.
Source Data Fig. 4 Statistical source data Fig. 4.
Source Data Fig. 5 Statistical source data Fig. 5.
Source Data Fig. 6 Statistical source data Fig. 6.
Source Data Fig. 6 Western blots Fig. 6.
Source Data Fig. 7 Statistical source data Fig. 7.
Source Data Fig. 8 Statistical source data Fig. 8.
Source Data Extended Data Fig. 1 Statistical source data Extended Data Fig. 1.
Source Data Extended Data Fig. 2 Statistical source data Extended Data Fig. 2.
Source Data Extended Data Fig. 4 Statistical source data Extended Data Fig. 4.
Source Data Extended Data Fig. 5 Statistical source data Extended Data Fig. 5.

## Code availability

The code used to analyze lipidomic data in this study is available in the GitHub repository for this paper (https://github.com/brunetlab).
