# Author Correction: Lipid droplets and peroxisomes are co-regulated to drive lifespan extension in response to mono-unsaturated fatty acids

**DOI:** 10.1038/s41556-023-01220-x

**Published:** 2023-08-11

**Authors:** Katharina Papsdorf, Jason W. Miklas, Amir Hosseini, Matias Cabruja, Christopher S. Morrow, Marzia Savini, Yong Yu, Carlos G. Silva-García, Nicole R. Haseley, Luke Meraz Murphy, Pallas Yao, Elisa de Launoit, Scott J. Dixon, Michael P. Snyder, Meng C. Wang, William B. Mair, Anne Brunet

**Affiliations:** 1 Department of Genetics, Stanford University, Stanford, CA USA; 2 Department of Molecular Metabolism, Harvard T. H. Chan School of Public Health, Boston, MA USA; 3 Department of Molecular and Human Genetics, Huffington Center on Aging, Baylor College of Medicine, Houston, TX USA; 4 Department of Biology, Stanford University, Stanford, CA USA; 5 Glenn Laboratories for the Biology of Aging, Stanford University, Stanford, CA USA; 6 Wu Tsai Institute of Neurosciences, Stanford University, Stanford, CA USA; 7 Present Address: State Key Laboratory of Cellular Stress Biology, School of Life Sciences, Faculty of Medicine and Life Sciences, Xiamen University, Xiamen, China; 8 Present Address: Janelia Research Campus, Howard Hughes Medical Institute, Ashburn, VA USA

**Keywords:** Cell biology, Genetics

Correction to: *Nature Cell Biology* https://www.nature.com/articles/s41556-023-01136-6. Published online 1 May 2023.

In the version of the article originally published, in the Extended Data Fig. 2a legend, the Methods “*C. elegans* and bacteria strains” subsection, the Nature Portfolio Reporting Summary and Supplementary Table 1, a *C. elegans* strain was inadvertently referred to as WBM1369 *lpin-1(sta10[lpin-1::GFP])*. The correct name is ABR339 *lpin-1(wbm76[lpin-1::GFP]*). We sincerely apologize for this error. This has been corrected in the HTML and PDF versions of the article.

