# Supplementary material for: Lipid droplets and peroxisomes are co-regulated to drive lifespan extension in response to mono-unsaturated fatty acids
Source: Nat Cell Biol. 2023 May 1;25(5):672–84. doi: 10.1038/s41556-023-01136-6 (PMC10185472; doi:10.1038/s41556-023-01136-6)
Supplement: Supplementary file 1 — Reporting Summary [file 41556_2023_1136_MOESM1_ESM.pdf]

nature portfolio

Corresponding author(s): Anne Brunet
Last updated by author(s): 08/03/2023

Reporting Summary

Nature Portfolio wishes to improve the reproducibility of the work that we publish. This form provides structure for consistency and transparency in reporting. For further information on Nature Portfolio policies, see our Editorial Policies and the Editorial Policy Checklist.

Statistics

For all statistical analyses, confirm that the following items are present in the figure legend, table legend, main text, or Methods section.

n/a	Confirmed	
☐	☒	The exact sample size (*n*) for each experimental group/condition, given as a discrete number and unit of measurement
☐	☒	A statement on whether measurements were taken from distinct samples or whether the same sample was measured repeatedly
☐	☒	The statistical test(s) used AND whether they are one- or two-sided<br>*Only common tests should be described solely by name; describe more complex techniques in the Methods section.*
☒	☐	A description of all covariates tested
☐	☒	A description of any assumptions or corrections, such as tests of normality and adjustment for multiple comparisons
☐	☒	A full description of the statistical parameters including central tendency (e.g. means) or other basic estimates (e.g. regression coefficient) AND variation (e.g. standard deviation) or associated estimates of uncertainty (e.g. confidence intervals)
☒	☐	For null hypothesis testing, the test statistic (e.g. *F*, *t*, *r*) with confidence intervals, effect sizes, degrees of freedom and *P* value noted<br>*Give P values as exact values whenever suitable.*
☒	☐	For Bayesian analysis, information on the choice of priors and Markov chain Monte Carlo settings
☒	☐	For hierarchical and complex designs, identification of the appropriate level for tests and full reporting of outcomes
☐	☒	Estimates of effect sizes (e.g. Cohen's *d*, Pearson's *r*), indicating how they were calculated

Our web collection on statistics for biologists contains articles on many of the points above.

Software and code

Policy information about availability of computer code

Data collection	NIS-Elements software (version AR 4.30.02)<br>Zen blue edition (version 3.0)<br>BioSorter FlowPilot (version III)<br>MassHunter GC/MS Acquisition (version 10.0.368)
Data analysis	Data analysis was performed using<br>R (version 3.6.3) using the packages<br>forcats (v0.5.1)<br>stringr (v1.4.0)<br>dplyr (v1.0.5)<br>purrr (0.3.4)<br>readr (v1.4.0)<br>tidyr (v1.1.3)<br>tibble (v3.1.1)<br>ggplot2 (v3.3.3)<br>tidyverse (v1.3.1)<br>stringr (v1.4.0)<br>data.table (v1.13.6)<br>ggpubr (v0.4.0.999)<br>RColorBrewer (v1.1-3)<br>pheatmap (v1.0.12)

The code used to analyze lipidomic data in the current study are available in the Github repository for this paper (https://github.com/brunetlab).

Other analysis tools used include:
Fiji (version 2.0.0),
Enhanced ChemStation (version F.01.03.2357)
LipidSearch (version 4.1)
Prism (Version 9) and WormEnrichR August 1st, 2018 (https://maayanlab.cloud/WormEnrichr/)

For manuscripts utilizing custom algorithms or software that are central to the research but not yet described in published literature, software must be made available to editors and reviewers. We strongly encourage code deposition in a community repository (e.g. GitHub). See the Nature Portfolio guidelines for submitting code & software for further information.

Data

Policy information about availability of data

All manuscripts must include a data availability statement. This statement should provide the following information, where applicable:
- Accession codes, unique identifiers, or web links for publicly available datasets
- A description of any restrictions on data availability
- For clinical datasets or third party data, please ensure that the statement adheres to our policy

Raw lipidomic files are available at Metabolomics Workbench (https://www.metabolomicsworkbench.org/) under study ID ST002504. All other lipidomic files are available in the Github repository for this paper (https://github.com/brunetlab). Source Data of experiments, replicates and statistics are provided in the Source Data. For the genes used in this study, the annotated wormbase name was used (www.wormbase.org)

Human research participants

Policy information about studies involving human research participants and Sex and Gender in Research.

Reporting on sex and gender	NA
Population characteristics	NA
Recruitment	NA
Ethics oversight	NA

Note that full information on the approval of the study protocol must also be provided in the manuscript.

Field-specific reporting

Please select the one below that is the best fit for your research. If you are not sure, read the appropriate sections before making your selection.

☒ Life sciences ☐ Behavioural & social sciences ☐ Ecological, evolutionary & environmental sciences

For a reference copy of the document with all sections, see nature.com/documents/nr-reporting-summary-flat.pdf

Life sciences study design

All studies must disclose on these points even when the disclosure is negative.

Sample size

Sample size was based on previous experiments and prior literature using similar experimental paradigms:

Reference for SRS phenotype: Mutlu, A. S., Chen, T., Deng, D. & Wang, M. C. Label-Free Imaging of Lipid Storage Dynamics in Caenorhabditis elegans using Stimulated Raman Scattering Microscopy. J Vis Exp, doi:10.3791/61870 (2021).

Reference for lipid droplet phenotype (similar to Oil Red O quantification), lifespan assays, gas chromatography assays and RNA quantifications: Han, S. et al. Mono-unsaturated fatty acids link H3K4me3 modifiers to C. elegans lifespan. Nature 544, 185-190, doi:10.1038/nature21686 (2017). Sample sizes for peroxisome quantification were based on lipid droplet quantifications.

Reference for liquid chromatography MS/MS assays: Cabruja, M. et al. In-depth triacylglycerol profiling using MS3 Q-Trap mass spectrometry. Anal Chim Acta, 2021 Nov 1;1184:339023. doi: 10.1016/j.aca.2021.339023. Epub 2021 Sep 3.

No statistical method was used to predetermine sample size as indicated in Materials and Methods (Statistics section). We have clearly indicated cases where samples from independent experiments were combined, and have included all combined and non-combined data (and associated statistics) in the data tables.

Data exclusions

For automated peroxisome counting images were excluded based on the following pre-established criteria. In cases with no peroxisomal

Data exclusions	localized fluorophore and only cytosolic fluorophore, the particle analysis plugin squassh failed to count 0 particles but instead counted thousand particles. These failures to count peroxisome particles occurred very rarely (less than 5% of all images) and were mostly restricted to the conditions with depleted peroxisome import (prx-5 RNAi).<br>In lifespan assays, we excluded plates based on the following pre-established criteria: 1) growth of bacteria that were not originally seeded on the plate and fungal growth. All other data was included in the study.
Replication	Key experiments were replicated three times independently. All data in this manuscript was replicated at least two times independently.
Randomization	Worms were randomly distributed into treatment and control groups. Lifespan assays and organelle imaging was performed randomized. Lipidomic sample preparation and mass spectrometry was performed randomized.
Blinding	The blinding information for each experiment and the corresponding replicates is listed in Source Data. Experiments related to the following figure panels were performed blinded: Figure 1e, 1f, 1g, 1h, 2b, 2c, 2f, 3b, 3c, 3d, 3e, 3g, 3h, 3i, 3j, 4b, 4c, 4d, 4e, 4f, 6i, 7c, 7d, 7e, 7f, 8c, 8d, 8e, 8f, 8g, 8h, 8i, 8j, 8k, Extended Data Figure 1a, 1b, 1h, 1i, 1j, 1k, 1l, 1n, 2e, 2g, 2h, 4b, 4c, 5a, 5b, 5c, 5d, 5e, 5f, 5i, 5n, 5o, 5p, 5q.The following experiments were not performed blinded: SRS experiments (Figure 1b, 1c, 1d, Extended Data Figure 1e, 1f). Lifespan assays to test supplementation with oleic acid or elaidic acid (Figure 2d) were not blinded because the difference between fatty acid plates can be visually distinguished. However, for lifespan assays with both fatty acid supplementation (e.g. oleic acid) and RNAi interventions the bacteria were blinded. Additionally, for lipid droplet imaging upon fatty acid supplementation, the worms were blinded prior to imaging. The preparation of samples for lipidomic quantification (Figure 5b-e, 5g, Extended Data Figure 4a), MDA level quantification (Figure 6a, 6c, 6f-h, Extended Data Figure 5g), GC/MS quantification (Extended Data Figure 1c, 1d, 1m) and RNA level quantification (Extended Data Figure 2c) was not performed blinded. Figure panels 6d-e, 8a, Extended Data Figure 4d, 5j, 5k, 5l, 5m were not performed blinded.

Reporting for specific materials, systems and methods

We require information from authors about some types of materials, experimental systems and methods used in many studies. Here, indicate whether each material, system or method listed is relevant to your study. If you are not sure if a list item applies to your research, read the appropriate section before selecting a response.

Materials & experimental systems

n/a	Involved in the study
☐	☒ Antibodies
☒	☐ Eukaryotic cell lines
☒	☐ Palaeontology and archaeology
☐	☒ Animals and other organisms
☒	☐ Clinical data
☒	☐ Dual use research of concern

Methods

n/a	Involved in the study
☒	☐ ChIP-seq
☐	☒ Flow cytometry
☒	☐ MRI-based neuroimaging

Antibodies

Antibodies used	The following antibodies were used in this study:<br>anti 4-HNE (Millipore Sigma, AB5605, lot#, 3574572, diluted 1:2000)<br>anti alpha-Tubulin (Millipore Sigma, T6074, lot# 0000093770, diluted 1:10 000)<br>anti goat-HRP (Calbiochem, 401515, lot# D00096831, diluted 1:10 000)<br>anti mouse-HRP (Calbiochem, 401215, lot#D00157542, 1:10 000)<br>anti rabbit-GFP (Abcam, ab6556, lot# GF3351352-1, 1:100)
Validation	All the antibodies used in this work are commercially available and have been published/cited:<br>https://www.citeab.com/antibodies/223716-ab5605-anti-4-hydroxynonenal-antibody?des=cf4ae4f41950c513<br>https://www.citeab.com/antibodies/2304938-t6074-monoclonal-anti-alpha-tubulin-antibody-produce?des=87a6f1d3abab97ee<br>https://www.citeab.com/antibodies/10901783-401515-rabbit-anti-goat-igg-h-l-chain-specific-p?des=2453a0952a4bd2cd<br>https://www.citeab.com/antibodies/10905348-401215-goat-anti-mouse-igg-h-l-chain-specific-pero?des=b3748f265c8dfff1<br>https://www.citeab.com/antibodies/732940-ab6556-anti-gfp-antibody?des=a4830a1239bf8d84

Animals and other research organisms

Policy information about studies involving animals; ARRIVE guidelines recommended for reporting animal research, and Sex and Gender in Research

Laboratory animals	Hermaphrodites and males of the following ages were used in this study: Young adult (adult day 1), middle-aged (adult day 5 and 6), old (adult day 8 and 10). For lifespan assays the worms were monitored across their whole lifespan.<br>The following C. elegans strains were used in this study<br>ABR161: hjls37 [vha-6p::mRFP-SKL + Cbr-unc-119(+)]; Idrls1 [dhs-3p::dhs-3::GFP + unc-76(+)]<br>N2: Wild type<br>FX02100: tm2100<br>LIU1: Idrls1 [dhs-3p::dhs-3::GFP + unc-76(+)]<br>LIU2: Idrls2 [plinp::plin-1::mCherry + unc-76(+)]. Note: The plin-1 gene (W01A8.1) was formerly annotated mdt-28 in wormbase. We now use the current annotation of plin-1 for W01A8.1.

VC3933: R01B10.6 (gk5008[loxP + myo-2p::GFP::unc-54 3'UTR + rps-27p::neoR::unc-54 3'UTR + loxP]) V
VS10: hjls37 [vha-6p::mRFP-SKL + Cbr-unc-119(+)]
VS15: hjls8 [ges-1p::GFP-SKL]
WBM1140: wbmIs65 [eft-3p::3XFLAG::dpy-10 crRNA::unc-54 3'UTR]
WBM1177: wbmIs81 [eft-3p::3XFLAG::GFP::SKL::unc-54 3'UTR, *wbmIs65]
ABR339: lpin-1 (wbm76[lpin-1::GFP])

Wild animals	No wild animals were used in this study.
Reporting on sex	All experiments were carried out in hermaphrodites, with the exemption of experiments related to Fig. 1g and Extended Data Fig. 1g which were carried out in males. Sex was assigned using morphological differences (male tail, presence of oocytes, uterus and vulva) at adult day 1. We have indicated in the methods which sex was used for the experiment. Hermaphrodites are the predominant sex, with an occurrence of 99.5 to 99.8%, which makes experiments using males more challenging.
Field-collected samples	No field-collected samples were used in this study.
Ethics oversight	No ethical approval or guidance was required.

Note that full information on the approval of the study protocol must also be provided in the manuscript.

Flow Cytometry

Plots

Confirm that:

☒ The axis labels state the marker and fluorochrome used (e.g. CD4-FITC).

☒ The axis scales are clearly visible. Include numbers along axes only for bottom left plot of group (a 'group' is an analysis of identical markers).

☐ All plots are contour plots with outliers or pseudocolor plots.

☒ A numerical value for number of cells or percentage (with statistics) is provided.

Methodology

Sample preparation	Adult day 1 GFP fluorescence LIU1 worms fed on control (empty vector) RNAi were washed off plates with M9 buffer and resuspended in M9 for sorting.
Instrument	BioSorter (Union Biometrica)
Software	BioSorter FlowPilot (version III)
Cell population abundance	We sorted adult worms based on GFP fluorescence intensity. The top and bottom 10% were sorted and we verified with a fluorescence dissection microscope that the sorting was successful.
Gating strategy	To exclude bacteria, debris and eggs from the sorting worms were gated based on time of flight and extinction. All worms within that population were GFP positive. The 10% highest and lowest GFP fluorescent worms were sorted on plates for subsequent analysis. All gates are shown in Extended Data Fig. 3a.

☒ Tick this box to confirm that a figure exemplifying the gating strategy is provided in the Supplementary Information.
