# Supplementary material for: Lipid droplets and peroxisomes are co-regulated to drive lifespan extension in response to mono-unsaturated fatty acids
Source: Nat Cell Biol. 2023 May 1;25(5):672–84. doi: 10.1038/s41556-023-01136-6 (PMC10185472; doi:10.1038/s41556-023-01136-6)
Supplement: Source Data Fig. 6 — Western blots Fig. 6. [file 41556_2023_1136_MOESM9_ESM.pdf]

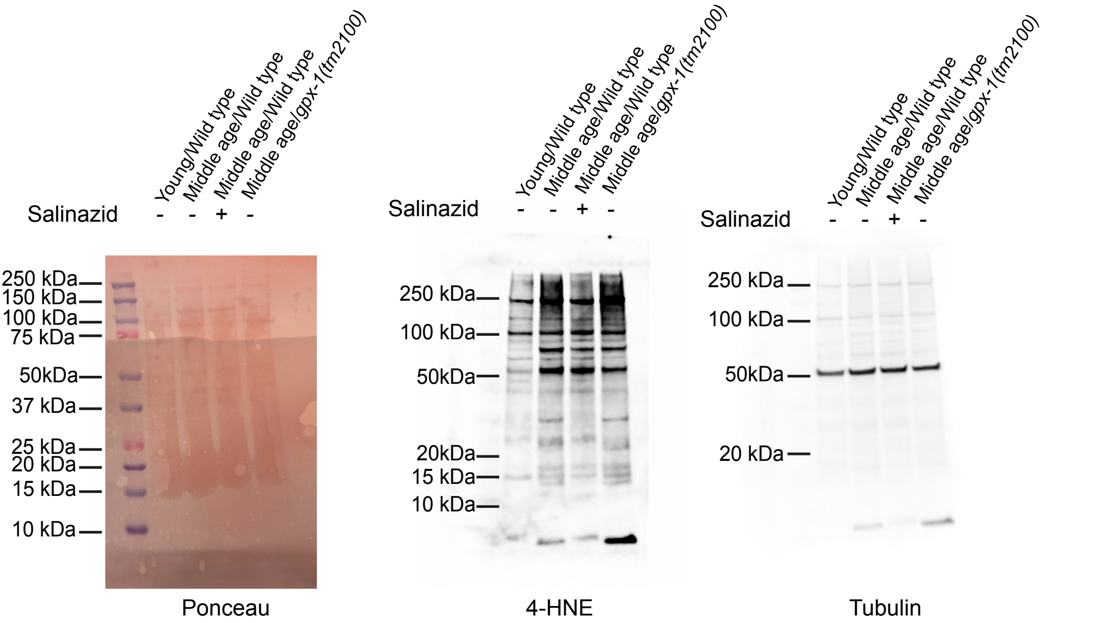

Young/Wild type
Middle age/Wild type
Middle age/Wild type
Middle age/gpx-1(tm2100)
Salinazid - - + -
250 kDa
150 kDa
100 kDa
75 kDa
50kDa
37 kDa
25 kDa
20 kDa
15 kDa
10 kDa
Ponceau
Young/Wild type
Middle age/Wild type
Middle age/Wild type
Middle age/gpx-1(tm2100)
Salinazid - - + -
250 kDa
100 kDa
50kDa
20kDa
15 kDa
10 kDa
4-HNE
Young/Wild type
Middle age/Wild type
Middle age/Wild type
Middle age/gpx-1(tm2100)
Salinazid - - + -
250 kDa
100 kDa
50kDa
20 kDa
Tubulin
